# Anti-Staphy Peptides Rationally Designed from Cry10Aa Bacterial Protein

**DOI:** 10.1021/acsomega.3c07455

**Published:** 2024-06-19

**Authors:** Thuanny Borba Rios, Mariana Rocha Maximiano, Fabiano Cavalcanti Fernandes, Gabriella Cavalcante Amorim, William Farias Porto, Danieli Fernanda Buccini, Valentina Nieto Marín, Gabriel Cidade Feitosa, Carlos Daniel Pereira Freitas, Juliana Bueno Barra, Antonio Alonso, Maria Fátima Grossi de Sá, Luciano Morais Lião, Octávio Luiz Franco

**Affiliations:** † S-Inova Biotech, Programa de Pós-Graduação em Biotecnologia Universidade Católica Dom Bosco, Av. Tamandaré, 6000—Jardim Seminario, Campo Grande, MS 79117-900, Brazil; ‡ Centro de Análises Proteômicas e Bioquímicas, Programa de Pós-Graduação em Ciências Genômicas e Biotecnologia Universidade Católica de Brasília, St. de Grandes Áreas Norte 916—Asa Norte, Brasília, DF 70790-160, Brazil; § Embrapa Recursos Genéticos e Biotecnologia, Parque Estação Biológica, PqEB, Av. W5 Norte—Asa Norte, Brasília, DF 70770-917, Brazil; ∥ Porto Reports, Brasília, DF 70770-917, Brazil; ⊥ Laboratório de RMN, Instituto de Química, Universidade Federal de Goiás, Goiânia, GO 74690-900, Brazil; # Pós-Graduação em Patologia Molecular, Universidade de Brasília, Campus Darcy Ribeiro, Brasília, DF 70910-900, Brazil; ∇ Instituto de Física, Universidade Federal de Goiás, Goiânia, GO 74690-900, Brazil

## Abstract

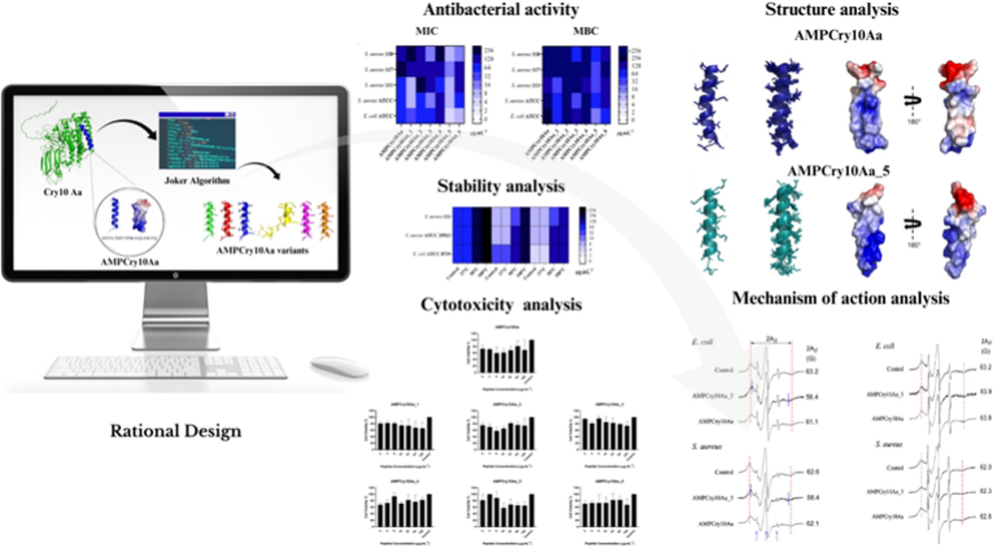

Bacterial infections pose a significant threat to human health, constituting a major challenge for healthcare systems. Antibiotic resistance is particularly concerning in the context of treating staphylococcal infections. In addressing this challenge, antimicrobial peptides (AMPs), characterized by their hydrophobic and cationic properties, unique mechanism of action, and remarkable bactericidal and immunomodulatory capabilities, emerge as promising alternatives to conventional antibiotics for tackling bacterial multidrug resistance. This study focuses on the Cry10Aa protein as a template for generating AMPs due to its membrane-penetrating ability. Leveraging the Joker algorithm, six peptide variants were derived from α-helix 3 of Cry10Aa, known for its interaction with lipid bilayers. In vitro, antimicrobial assays determined the minimum inhibitory concentration (MIC) and minimum bactericidal concentration (MBC) required for inhibiting the growth of *Staphylococcus aureus*, *Escherichia coli*, *Acinetobacter baummanii*, *Enterobacter cloacae*, *Enterococcus facallis*, *Klebsiella pneumonia*, and *Pseudomonas aeruginosa*. Time-kill kinetics were performed using the parental peptide AMPCry10Aa, as well as AMPCry10Aa_1 and AMPCry10Aa_5, against *E. coli* ATCC, *S. aureus* 111 and *S. aureus* ATCC strains showing that AMPCry10Aa_1 and AMPCry10Aa_5 peptides can completely reduce the initial bacterial load with less than 2 h of incubation. AMPCry10Aa_1 and AMPCry 10Aa_5 present stability in human serum and activity maintenance up to 37 °C. Cytotoxicity assays, conducted using the MTT method, revealed that all of the tested peptides exhibited cell viability >50% (IC50). The study also encompassed evaluations of the structure and physical-chemical properties. The three-dimensional structures of AMPCry10Aa and AMPCry10Aa_5 were determined through nuclear magnetic resonance (NMR) spectroscopy, indicating the adoption of α-helical segments. Electron paramagnetic resonance (EPR) spectroscopy elucidated the mechanism of action, demonstrating that AMPCry10Aa_5 enters the outer membranes of *E. coli* and *S. aureus*, causing substantial increases in lipid fluidity, while AMPCry10Aa slightly increases lipid fluidity in *E. coli*. In conclusion, the results obtained underscore the potential of Cry10Aa as a source for developing antimicrobial peptides as alternatives to conventional antibiotics, offering a promising avenue in the battle against antibiotic resistance.

## Introduction

Bacterial infections, due to their rising occurrence and dissemination, pose a hazard to human health and a severe concern for healthcare systems.^1^ Emerging and re-emerging infectious diseases have been identified as one of the greatest public health issues in the last three decades, and despite modern health care, bacterial infectious diseases remain one of the leading causes of global mortality.^2^

*Staphylococcus aureus* is a Gram-positive opportunistic pathogen responsible for several diseases, ranging from skin infections and abscesses to much more severe endocarditis, osteomyelitis, pneumonia, meningitis, and sepsis.^3−5^ Resistance to currently used antibiotics, such as methicillin, vancomycin, daptomycin, and linezolid, is a serious issue in staphylococcal infection treatment.^6−8^ Antimicrobial resistance is a natural phenomenon produced by exposure of microorganisms to antibiotic molecules and has been treated as one of the greatest threats to public health in the 21st century.^9,10^ Selective pressure from the use of different antibiotics gives microorganisms a greater chance of surviving and replicating. This might be related to the selection of naturally/intrinsically resistant bacteria or those that have acquired antibiotic-resistant traits.^11,12^ These multidrug-resistant (MDR) species are becoming a growing concern and include species such as *Staphylococcus aureus*.^13−15^

Antimicrobial peptides (AMPs) have been identified as promising alternatives to antibiotics frequently used for treating bacterial infections. AMPs are small oligo peptides, containing up to 50 amino acid residues.^16−18^ AMPs have a broad range of lytic activities, although they prefer to target lipid membranes, for which they have a higher affinity than the aqueous environment. Electrostatic and hydrophobic interactions drive their adsorption onto membranes, causing modifications to structure in both the peptide and the lipid membrane.^19^ These peptides have a wide range of activities against Gram-negative and -positive bacteria, fungi, parasites, and viruses. AMP production can be performed by chemical synthesis^18,20−22^ or using recombinant expression systems.^23−26^ AMP synthesis also aims to achieve greater selectivity and a decrease in hemolytic activity or cytotoxicity for the host cells.

The development of new synthetic AMPs, by amino acid substitution, to improve the activity of natural peptides can increase stability and resistance to proteolytic degradation, in addition to increasing antimicrobial activity. Among all the characteristics described, shorter AMPs are preferred, in order to reduce production costs, and many of these exhibit antibacterial potential against clinical isolates.^27,28^ These artificial AMP sources are useful for modifying and creating new synthetic AMPs.^29^ Rational design emerges as an alternative that may or may not use prior information about the three-dimensional structures of proteins or peptides to make changes.

Furthermore, without the need for a model sequence, de novo computational approaches create AMP sequences with amino acid position and frequency preferences that can ensure properties such as charge, amphipathicity, and structure.^30^ This approach has enabled the generation of many sequences with a wide range of amino acid arrangements, tridimensional structures, and mechanisms of action.^31^ The de novo model has been used to develop a growing variety of tools, including linguistic models.^32^ AMPs may be constructed through formal language that comprises vocabulary (e.g., amino acid residues) and rules (e.g., amino acid patterns). As a result, it is proposed that by using this “grammar” approach, AMPs might operate more specifically by detecting targets within cells or acting specifically on bacterial membranes. This concept has recently been expanded by associating the discovery of amino acid patterns in public databases with their subsequent insertion into a peptide sequence with the goal of producing optimal AMPs.^27^

Despite the great potential that AMPs have in combating microorganisms, the search for new molecules and methods continues. Thus, the study of the antimicrobial potential of crystalline proteins, such as Cry10Aa, produced by *Bacillus thuringiensis* has been growing over time. These proteins, as well as some of their fragments, obtained through proteolysis have antimicrobial properties.^33,34^

Cry proteins are insecticidal crystalline proteins generated at the beginning of the sporulation phase and throughout the stationary growth phase of *Bacillus thuringiensis* (Bt) bacteria.^35^ These crystals are divided into three groups: (i) α- and β-exotoxins, (ii) δ-endotoxins (Cry and Cyt proteins), and (iii) VIP proteins “Vegetative Insecticidal Protein”.^36^

**Figure 1:**
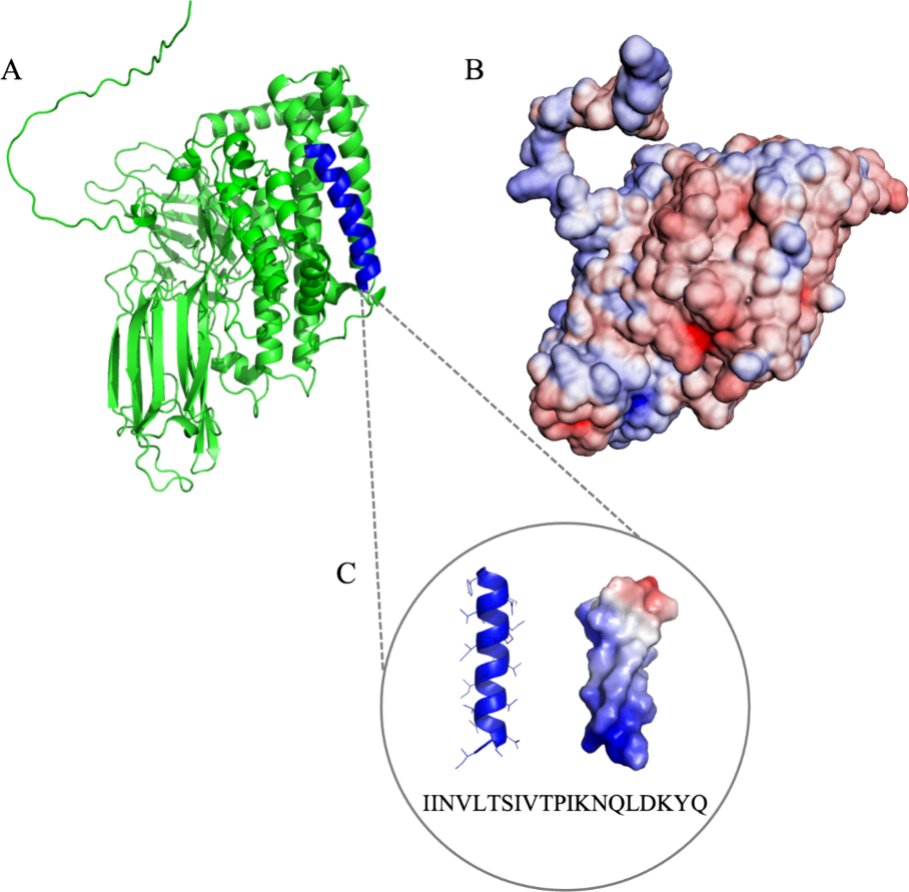
Cry10Aa protein molecular model in ribbons (A) and electrostatic surface (B) with anionic regions in red and cationic regions in blue, and 20 amino acid sequence of the α-helix 3 in blue, which was used as a template sequence for the present project (C). Both were visualized by PyMOL software v. 1.8.

Cry toxins share highly conserved tertiary structures, which are composed of three domains (Figure 1A).^37^ Domain I is located in the N-terminal portion.^38^ Domain II is located between domains I and III, in the central portion of the protein.^37^ Domain III, on the other hand, includes the C-terminus of most Cry toxins.^38,39^ Domain I is made up of 7–8 α-helices, with a centrally positioned hydrophobic α-helix 5, shows similarities to the pore-forming domains in other bacterial toxins and has proven to be involved in membrane insertion and pore formation.^40^ Among the Cry proteins already described in the literature, Cry10Aa shows characteristics that allow potential use for antimicrobial peptide development. The cationic regions (Figure 1B) can be involved in antibacterial activity,^37,41^ including the α- helix 3 (Figure 1C). Lin et al.^42^ performed in silico studies demonstrating that the α-helix 3 of Cry8Aa has an α-helical structure and that it is properly inserted in lipid bilayers. In this model, α-helix 3 might generate intermonomer interactions without major domain I rearrangements, which is consistent with the available Cry toxins’ crystalline structure in solution. These data demonstrate the potential for membrane interaction that these sequences may have, even being detached from the domain, which is already defined as the domain responsible for membrane and pore formation interaction.

Therefore, in this study, we aimed to carry out modifications using bioinformatics tools in the sequence of the Cry10Aa protein, aiming to develop variants that can be applied as an alternative to antimicrobial agents in managing bacterial infections. Furthermore, we aimed to determine and analyze the three-dimensional structures by solution NMR spectroscopy of peptide AMPCry10Aa, cut from the α-helix portion of the Cry10Aa protein, and peptide AMPCry10Aa_5, which showed the most promising MIC results among the other variants generated by the Joker algorithm.

## Results and Discussion

### Development of Variants by Using Joker Software

The rational design of AMPs has shown great potential for the alternative use of natural molecules.^43^ The linguistic model for designing AMPs has gained attention in the recent decade since it recognizes sequences of amino acids as a formal language that can be represented by a set of grammatical rules.^32^ Thus, according to the linguistic model, each amino acid stands for a “word” that must be placed in the proper position for the sentence (sequence) to make sense. The principles were originally described by Loose et al.^32^ In this way, the Joker^27^ is a simple algorithm to use, in which it is only necessary to use a template sequence and a pattern.

**Figure 2:**
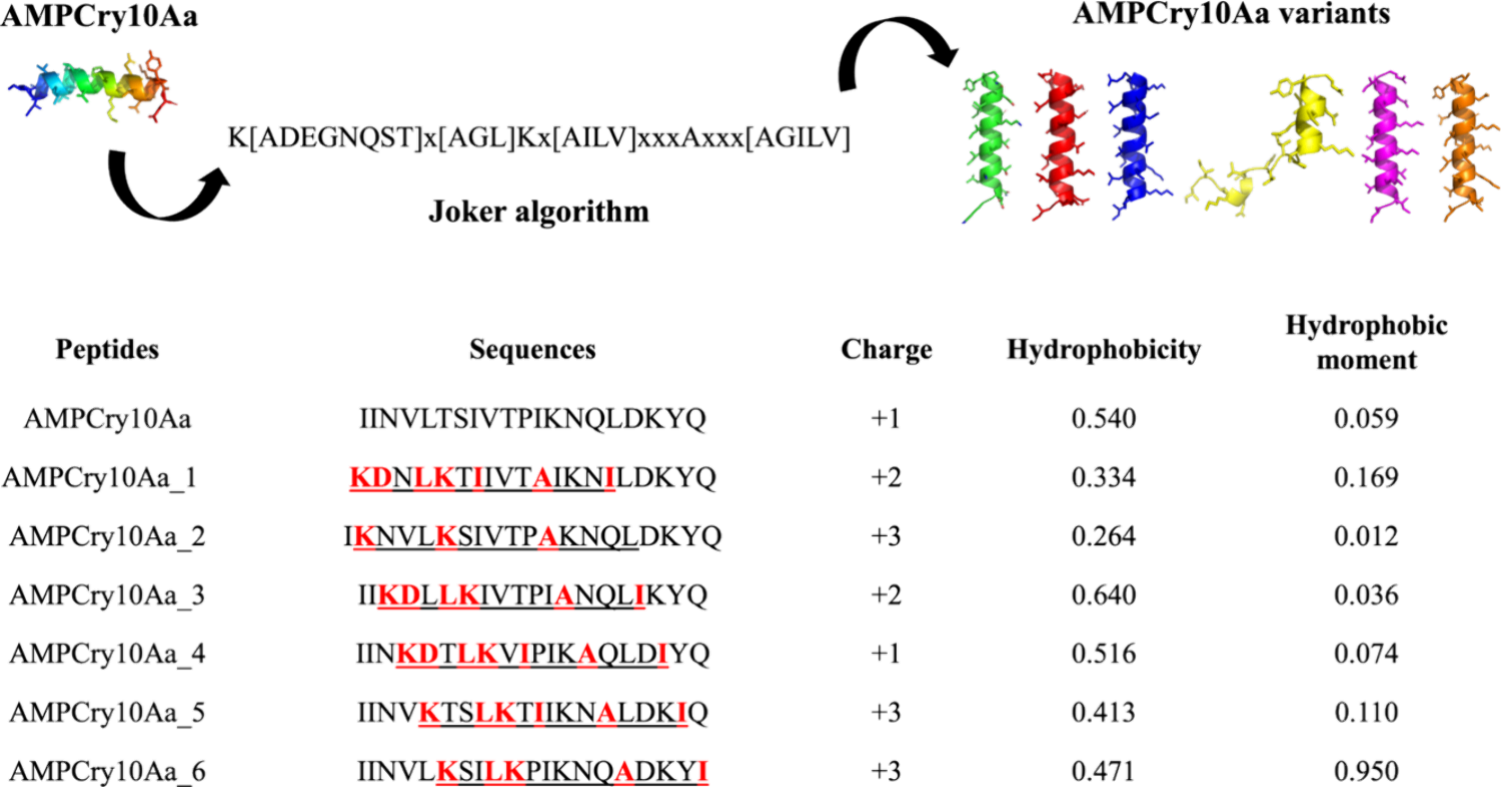
Variants generated by the Joker algorithm from the insertion of the pattern in the parental sequence and their respective sequences, with modifications highlighted in red, and physical–chemical characteristics.

Joker^27^ inserted the pattern (K-[ADEGNQST]-x-[AGL]-K-x-[AIL V]-x(3)-A-x(3)-[AGILV]) throughout the parental sequence, which resulted in the development of six variants (Figure 2). The charge of all variants, except for AMPCry10Aa_4, was greater than the charge of the parental sequence (Figure 2). We can see that they all have a positive charge as well as the parental sequence.

### Antibacterial and Time-Kill Kinetics Assays

**Figure 3:**
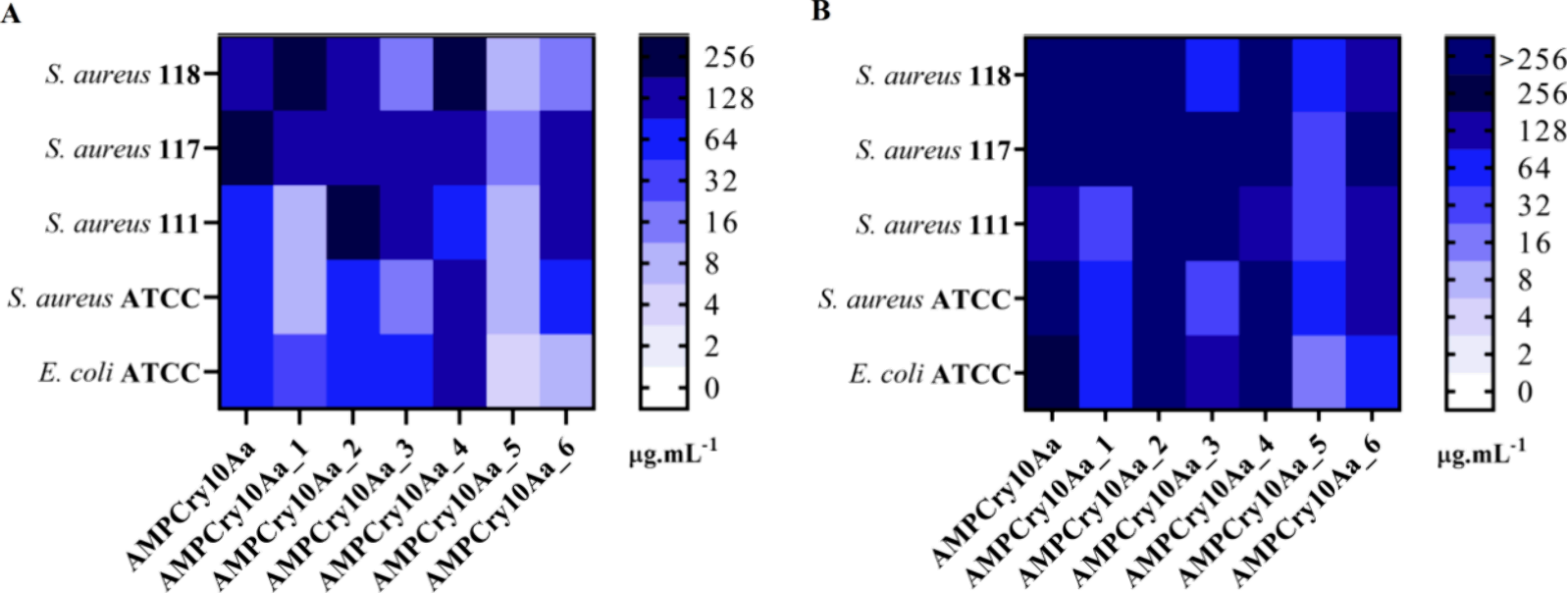
Heatmap of peptide functional analyses. Minimum inhibitory concentration (MIC) (A) and minimum bactericidal concentration (MBC) (B) analyses for parental peptide and its variants against *Escherichia coli* ATCC 8739 and *Staphylococcus aureus* ATCC 25922 and *Staphylococcus aureus* isolates. Values were expressed in μg·mL^–1^.

The generated variants, as well as the parental sequence, were tested against four *S. aureus* and one *E. coli* strains (Figure 3A, B), and 5 other ESKAPE strains to access a wider spectrum of pathogens (Figure S1). In general, all variants showed activity, inhibiting the growth of at least one bacterium. However, aiming for lower minimal inhibitory concentrations (MIC) and minimal bactericidal concentrations (MBC) values (<32 μg·mL^–1^), three of the seven sequences (parental and variants) showed promising results. Bioactive peptides encoded in protein sequences seem to be much more frequent in nature than initially imagined, and their biotechnological potential is still being investigated. We can therefore suggest that perhaps the α-helix 3 sequence is a peptide encrypted in the Cry10Aa protein. Encrypted peptides may be present in protein structures of high molecular mass, which are released under certain physiological conditions to exert their function.^44,45^ However, the possible AMPs developed here are unprecedented since peptides generated from crystalline toxins, such as Cry10Aa, do not yet exist.

MIC results showed that AMPCry10Aa_5 and AMPCry10Aa_6 were more active against *E. coli* ATCC 25922 (Gram-negative) and AMPCry10Aa_1 and AMPCry10Aa_3 were more active against *S. aureus* ATCC 8739 (Gram-positive). The other variants as well as AMPCry10Aa, showed the same activity for both strains. AMPCry10Aa_5 stood out the most, for MIC and MBC assays, followed by AMPCry10Aa_1 (Figure 3A, B). Interestingly, there are similarities between these sequences, including a higher percentage of isoleucine in both than any other amino acid (25% in variant 1 and 20% in variant 5), and a similar charge distribution, with a very well-defined hydrophobic portion interspersed with 4 basic amino acids (Figure 5). AMPCry10Aa_5 stands out from the others because its MIC and MBC values are lower for all the strains tested; its characteristics stand out from the AMPCry10Aa, as they have a higher charge, in addition to a greater hydrophobic moment (Figure 2), also indicating that the insertion of the dermaseptin B pattern, allied to these characteristics, contributed to lower MIC and MBC values. Besides, analyzing the NMR structure and the electrostatic surface (Figure 6), AMPCry10Aa_5 assumed an α-helical conformation in Val4-Ile19, exhibited high flexibility, and displayed a strongly cationic nature, indicating that electrostatic attraction may be vital to the process of its mechanism of action, working in conjunction with the hydrophobic interactions described by the hydrophobic face of the peptide. This interaction most likely results in the phospholipid bilayer disordering, which ultimately contributes to the satisfactory antibacterial activity displayed by the peptide. Furthermore, it is important to state that peptides have different activities for different strains of bacteria, and this is due to membrane compositions. The membrane compositions of different bacterial species vary, and even cells within a single species exhibit variations in their membrane composition.^46,47^

**Figure 4:**
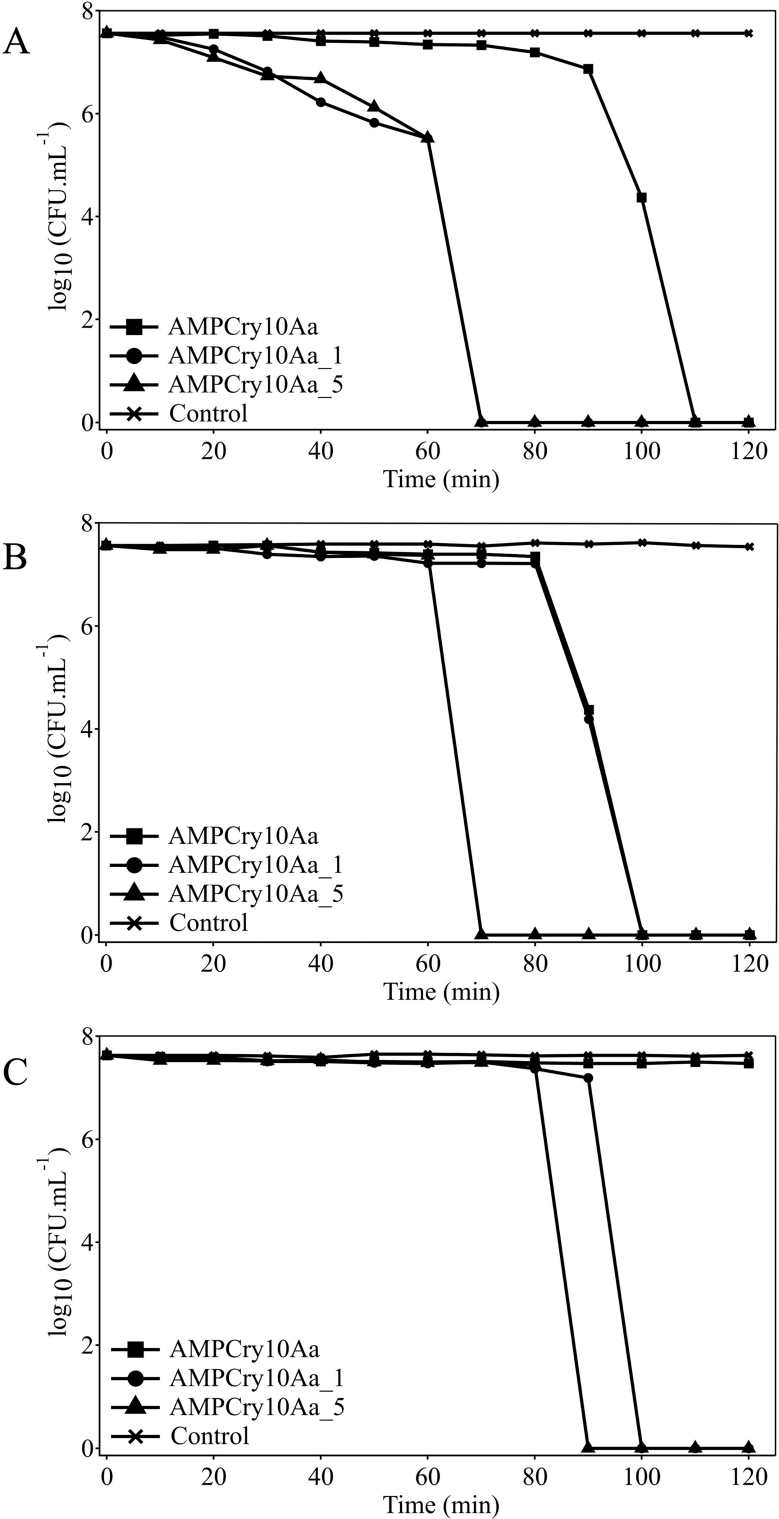
Time-kill kinetics of peptides AMPCry10Aa, AMPCry10Aa_1, and AMPCry10Aa_5 against *E. coli* ATCC (A), *S. aureus* 111 (B), and *S. aureus* ATCC (C) at 1× MBC. The bacterial strain growth in the absence of peptide was used as a growth control. The peptide was added at time 0, being monitored every 10 min until 2 h of incubation.

Regarding the time-kill kinetics assay, the time course of the bactericidal activity (time-kill) of AMPCry10Aa, AMPCry10Aa_1, and AMPCry10Aa_5 against *E. coli* ATCC, *S. aureus* 111 and *S. aureus* ATCC is shown in Figure 4. The time-kill curve shows that AMPCry10Aa_1 and AMPCry10Aa_5 can completely reduce the initial bacterial load in less than 2 h of incubation. AMPCry10Aa_5, in MBC, stands out from the others with an inhibition time of 70 min after incubation for both *E. coli* ATCC and *S. aureus* 111, while for *S. aureus* ATCC, the time for complete inhibition was 90 min.

Thus, we can consider that from a biotechnological point of view, an interesting discovery was made. The Cry10Aa protein can be a promising source in the search for antimicrobial molecules since its parental sequence showed activity without even going through the Joker algorithm. In the work by Lin et al.,^42^ in silico studies were carried out and the authors demonstrated that the α-helix 3 of Cry8Aa has an α-helical structure and that it is properly inserted in lipid bilayers. In this model, α-helix 3 may create intermonomer contacts without major domain I rearrangements, which is congruent with the known crystalline structure of Cry toxins in solution. These data demonstrate the potential for interaction with the membrane that these sequences may have, even being detached from the domain, which is already defined as the domain responsible for interaction with the membrane and pore formation. In addition, AMPCry10Aa_5 can be better exploited for future use in the fight against bacterial resistance.

### Peptide Stability and Activity Maintenance

The stability of AMPCry10Aa, AMPCry10Aa_1, and AMPCry10Aa_5 was tested in human serum to determine the time required to observe the complete degradation of the peptide (Figures S2–S4). Table 1 shows the peptide integrity percentages at different times. The intensity of the AMPCry10Aa peptide signal has already decreased to about 40% after 2 h and is no longer detectable after 12 h (Figure S2). Interestingly, AMPCry10Aa_1 and AMPCry10Aa_5 were more stable, respectively showing 60 and 40% of the initial peptide amount detected at 6 h after incubation and a trace at 12 h, being no longer detectable after 24 h (Figures S3 and S4).

**Table 1:** Percentage of Peptides Remaining in Human Serum after 24 h, Quantified by Reversed-Phase High-Performance Liquid Chromatography (RP-HPLC) Using a Reversed-Phase Venusil ASB C18 Column^a^^,^^b^

	relative peak area (%)^c^					
	0 h	2 h	4 h	6 h	12 h	24 h
AMPCry10Aa	92.2 ± 3.23	39.65 ± 2.44	18.6 ± 2.76	∼0	ND^d^	ND^d^
AMPCry10Aa_1	96.93 ± 0.51	81.81 ± 1.54	74.66 ± 1.98	58.96 ± 2.33	4.37 ± 1.32	ND^d^
AMPCry10Aa_5	92.83 ± 3.33	81.56 ± 2.21	68.77 ± 0.99	36.73 ± 1.84	8.11 ± 3.65	ND^d^

a Peptides were diluted to a final concentration of 256 μM by mixing the peptide with human serum and milk in a 1:5 ratio.

b Results represent the mean ± standard deviation (SD) from three replicates.

c Calculated by subtracting the relative area of treatment peaks from the initial control peak area.

d ND: not determined.

**Figure 5:**
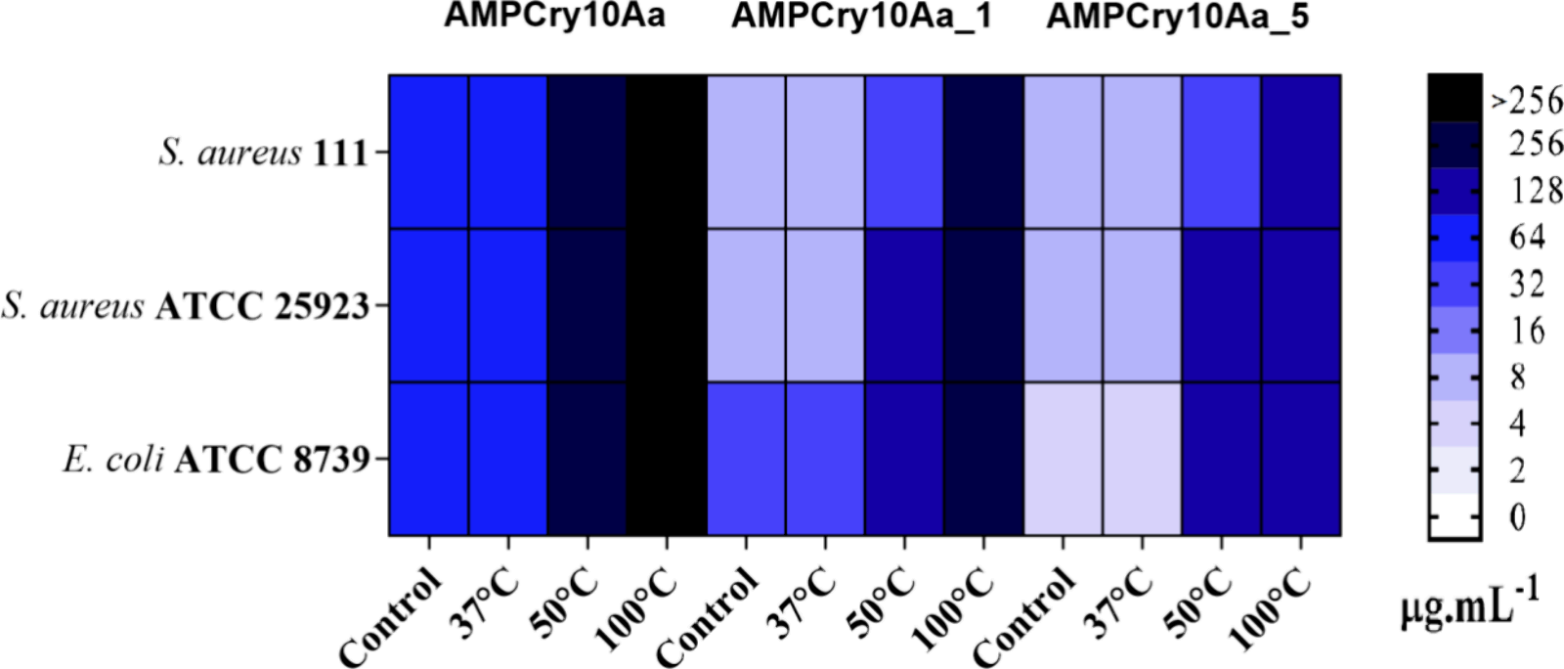
Heatmap of peptide antibacterial activities. Minimum inhibitory concentration (MIC) analyses for parental peptide and its variants against *Escherichia coli* ATCC 8739, *Staphylococcus aureus* ATCC 25922, and *Staphylococcus aureus* 111 after exposure for 30 min to 37, 50, and 100 °C temperatures. Values were expressed in μg·mL^–1^.

The temperature stability and antimicrobial activity maintenance were performed by an MIC assay after exposing the peptides to different temperatures (Figure 5). After exposure to 37 °C for 30 min, the antibacterial activity of the peptides remained unchanged. However, when exposed to 50 or 100 °C temperatures for 30 min, the parental peptide AMPCry10Aa lost the activity, indicating a possible degradation. Moreover, AMPCry10Aa_1 and AMPCry10Aa_5 also showed stability after exposure to 37 °C. However, both AMPCry10Aa_1 and AMPCry10Aa_5 did not tolerate temperatures of 50 and 100 well, slightly losing antimicrobial activity and presenting MIC values 4–8 times higher.

### Cytotoxicity Analysis

The parental peptide toxicity potential and the yielded variants generated were evaluated against murine macrophages (RAW 264.7 cells) (Figure S5). The peptides evaluated did not show cell viability below 50% (IC_50_), in any of the variants and concentrations tested, when compared to the control (100% viable cells).

### Variant Structural Analysis

**Figure 6:**
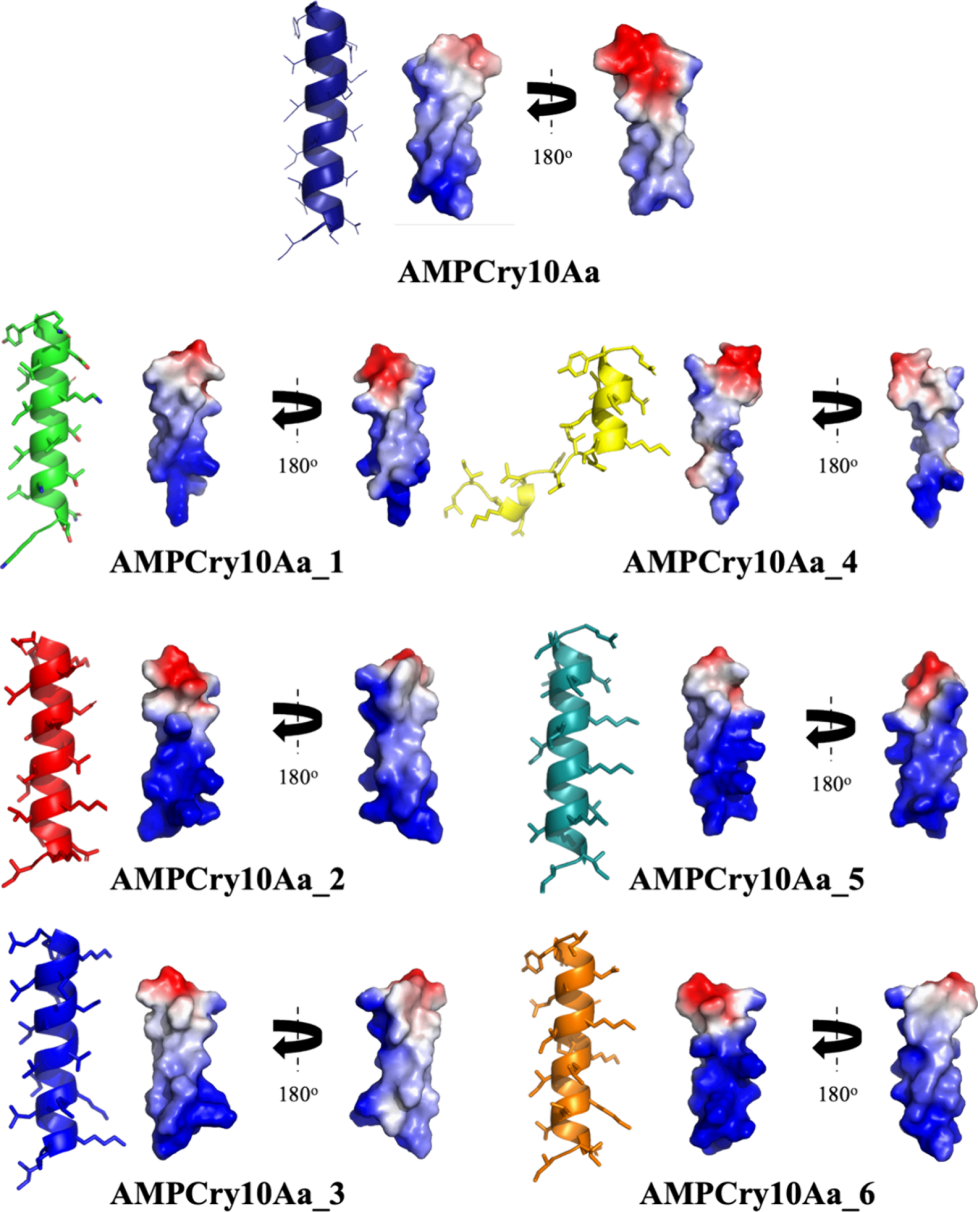
Predicted structures and electrostatic surface of the parental sequence and its variants. Both visualized by PyMOL software. In red are the anionic regions, in blue the cationic regions, and in white the neutral regions.

AMPs with an α-helical structure are generally more active in microbial membranes, as this structure helps them to insert themselves into cell membranes,^48,49^ also requiring an amphipathic structure with a well-defined hydrophobic sector. Helix formation allows an ideal spatial arrangement of amphipathic side chains for membrane insertion.^48,50,51^ The importance of this arrangement for the activity of α-helical AMPs is widely recognized because without it, the potent and wide-ranging antimicrobial activity would not be possible.^50^ Except for AMPCry10Aa_4, all variants, as well as the parental sequence, have these regions that are very well-defined. All yielded variants, as well as the parental sequence, all showed a predicted α-helix structure, apart from AMPCry10Aa_4, which demonstrated a helix break in the ^9^IPI^13^ region (Figure 6), probably due to the weak stability caused by a proline, which may have led to low activity levels (Figure 3A, B). The propensity of a helix to kink is strongly associated with the presence of proline residues within its sequence.^52−55^ Some studies have suggested that while many kinks are indeed linked to proline, others may be attributed to alternative mutational pathways^56^ and the presence of residues such as serine (Ser) and glycine (Gly) at the helical kink’s core.^57^ The hydrophobicity between the parental sequence and all variants remained very similar, except for AMPCry10Aa_2, which showed a lower value (Figure 2). Although the optimal hydrophobicity of a molecule varies according to its other characteristics, the hydrophobicity of most natural AMPs is around 50%. A small hydrophobic region can lead to an inability to insert into biological membranes to kill microorganisms, whereas a larger region can induce greater antimicrobial activity, as it favors interaction with phospholipid membranes and could increase sequence selectivity and stability. To some extent, increasing peptide hydrophobicity contributes to peptide molecules reaching the interface from an aqueous environment and improving antimicrobial action.^58^ However, increasing hydrophobicity too much can lead to a decrease in specificity for the bilipid layer which would reduce its capacity for antimicrobial action.^59^ There must be a balance of charges for more efficient activity to take place.^49^

With regard to the hydrophobic moment, the lowest values were obtained by AMPCry10Aa_2 and AMPCry10Aa_3, while AMPCry10Aa_6 was the one with the highest value, followed by AMPCry10Aa_1 and AMPCry10Aa_5. The hydrophobic moment is important because it concerns the measure of the amphipathicity of the α-helix, where the greater the hydrophobic moment, the greater the chance of interaction of the helix with the cell membrane, promoting a greater chance of antimicrobial activity.^60−63^

The hydrophobic moment of all variants increased, showing values higher than the parental sequence of 0.059, except for AMPCry10Aa_2 and AMPCry10Aa_3, which showed reduced hydrophobic moments of 0.012 and 0.036, respectively. The hydrophobicity of variants was reduced when compared to the parental sequence, which presents a value of 0.540, except for AMPCry10Aa_3 which presented a high hydrophobicity of 0.640. A lower value of hydrophobicity is known to reduce toxic effects against mammalian cells, including hemolysis.^58^

The charge of all variants, except for AMPCry10Aa_4, was higher than that of the parental sequence (Figure 2). Although neutral and anionic AMPs have been reported, positively charged peptides are generally more active because they are electrostatically attracted to microbial membranes that are negatively charged. There is broad agreement that these peptides target the instability of the cell membrane of Gram-negative and Gram-positive bacteria.^64^ Thus, electrostatic interactions with the negative charge of microbial cell surfaces result in pathogen membrane rupture. As a result, the positive charge of the cationic peptide appears to be an important factor to consider in the differentiation process between pathogen and host cells.^65^ The charges of AMPCry10Aa_2, AMPCry10Aa_3, and AMPCry10Aa_5 were the highest, which could be a positive characteristic and an indication of better activity.

For the parental sequence and variants 1–5, the α-helix structure was observed. However, AMPCry10Aa_4 was the only one that showed a loss of α-helix structure. Furthermore, AMPCry10Aa_4 was the only one that had fewer than 80% favorable regions in the Ramachandran Plot (Table S1). The models of all the generated variants (Figure 6), as well as the parental sequence, were analyzed by validation software.

### NMR Structural Analysis of AMPCry10Aa and AMPCry10Aa_5

The 3D structures of AMPCry10Aa and AMPCry10Aa_5 were determined by using NMR spectroscopy in 75 mM SDS-*d*_25_. The resonance assignments for the ^1^H nuclei were obtained by analyzing TOCSY and NOESY spectra, as reported by Wuthrich.^66^ The summary of structural statistics and quality analysis for the ensemble structures and the low-energy structure can be found in Table 2. The ^1^H–^1^H NOESY spectra analysis revealed 328 distance restraints for AMPCry10Aa and 291 restraints for AMPCry10Aa_5. These distance restraints and 34 dihedral angle restraints predicted by DANGLE in the CcpNMR Analysis program were used as input data in the ARIA protocol. We performed the structural calculation for 200 structures for each iteration, it0 until it8, starting with an extended structure until the MDSA protocol for energy minimization and to determine the secondary confirmation of the peptide. The ten lowest structures of the last iteration (it8) were refined in water, resulting in 10 structures used to represent the ensemble of AMPCry10Aa and AMPCry10Aa_5. The secondary structure was predicted using the phi (ϕ) and psi (ψ) angles obtained through the DANGLE algorithm, using experimental shifts of ^1^H_N_, ^1^H_α_, ^13^C_α_, and ^13^C_β_. Additional pertinent NMR data, including NOE connections, are summarized in Figure S6.

**Table 2:** NMR, Refinement Statistics, and Quality Validation for the Structures of AMPCry10Aa and AMPCry10Aa_5 in 75 mM SDS-*d*_25_ Micelles

	peptides	
NMR distance and dihedral constraints		
distance constraints	AMPCry10Aa	AMPCry10Aa_5
total restraints	328	291
intraresidue	218	197
sequential (|*i* – *j*| = 1)	58	69
short-range (2 ≤ |*i* – *j*| ≤ 3)	39	23
medium-range (4 ≤ |*i* – *j*| ≤ 5)	13	2
long-range (|*i* – *j*| > 5)	0	0
total dihedral angle restraints		
ϕ + ψ	34	34
structure statistics		
violations (mean and s.d.)		
distance constraints (Å)	0.0262 ± 0.0017	0.0231 ± 0.0018
dihedral angle constraint (deg)	0.0715 ± 0.1126	0.2961 ± 0.1393
max. dihedral angle violation (deg)	0.3092	0.4865
max. distance constraint violation (Å)	0.0297	0.0262
deviations from idealized geometry		
bond lengths (Å)	0.0042 ± 0.0019 × 10^–1^	0.0041 ± 0.0025 × 10^–1^
bond angles (deg)	0.4863 ± 0.0072	0.4922 ± 0.0186
impropers (deg)	1.3196 ± 0.1009	1.0615 ± 0.0793
average pairwise r.m.s. deviation^f^ (Å)		
backbone (second structure)^a^	0.4641 ± 0.1467	0.1393 ± 0.0467
heavy atoms (second structure)^a^	0.8822 ± 0.1767	0.6122 ± 0.1329
backbone (all residues)^b^	0.4647 ± 0.1468	1.1642 ± 0.5978
heavy atoms (all residues)^b^	0.8804 ± 0.1763	1.5300 ± 0.5580
Ramachandran plot (%)		
most favored regions	97.8	94.4
additional allowed regions	2.2	5.6
generously allowed regions	0	0
disallowed region	0	0
ProSA *Z*-score^c^	–0.20	–1.37
PROCHECK *G*-factors^d^	0.350	0.300
QMEAN^e^	0.41	1.14

a Pairwise r.m.s. deviation was calculated among 10 refined structures for residues in helical segment 2–20 to AMPCry10Aa and 4–19 to AMPCry10Aa_5.

b Pairwise r.m.s deviation was calculated among 10 refined structures for residues 1–20.

c *Z*-score value within the expected for NMR structures deposited in the PDB with a similar size and fold compared to AMPCry10Aa and AMPCry10Aa_5 calculated structures.

d *G*-factors score for dihedral angles and covalent forces of the main chain within the expected range for a reliable structure (>−0.05).

e QMEAN evaluates the model by comparing it with previously elucidated structures that are similar.

f All r.m.s. deviations were calculated by the CNS in refinement protocol.

For AMPCry10Aa, sequential HN–HN correlations were observed throughout the entire sequence, except for the Ile1 and Pro11 residues. An important feature of this peptide is the presence of Pro11, which breaks the sequential assignment in amidic hydrogen correlation. Regarding the H_β_-HN correlations, only the ones for Ile1 and Val4 were not observed. Concerning H_α_-HN type correlations, it was not possible to observe any for Ile1, Ile2, Asp3, Leu5, Thr6, 11Pro, and 19Tyr. NOEs of short (HN–HN, *i*, *i* + 2; H_α_–HN, *i*, *i* + 3 e H_α_–H_β_, *i*, *i* + 3) and medium distance (H_α_–HN, *i*, *i* + 4), characteristic of the α-helix, were observed for AMPCry10Aa, Figure S6a. The short distance NOE HN–HN *i*, *i* + 2 were observed among residues Ile2H-Val4H, Val4H-Thr6H, Leu5H-Ser7H, Thr6H-Ile8H, Ile8H-Thr10H, Gln15H-Asp17H, and 18LyH-Gln20H. For H_α_–HN *i*, *i* + 3 correlations, connections were observed among residues Asn3H_α_-Thr6H, Val4H_α_-Ser7H, Leu5H_α_-Ile8H, Thr6H_α_-Val9H, Va9H_α_-Ile12H, Ile12H_α_-Gln15H, Lys13H_α_-Leu16H, Asn14H_α_-Asp17H, Gln15H_α_-Lys18H, Leu16H_α_-Tyr19H, and Asp17H_α_-Gln20H. H_α_–H_β_*i*, *i* + 3 correlations were observed for Ile1H_α_-Val4H_β_, Ile2H_α_-Leu5H_β_, Thr6H_α_-Val9H_β_, Ser7H_α_-Thr10H_β_, Val9H_α_-Ile12H_β_, Ile12H_α_-Ala15H_β_, Asn14H_α_-Asp17H_β_, Gln15H_α_-Lys18H_β_, Leu16H_α_-Tyr19H_β_, and Asp17H_α_-Gln20H_β_. For H_α_–HN *i*, *i* + 2 correlations were observed between Asn3H_α_-Leu5H, Val4H_α_-Thr6H, Leu5H_α_-Ser7H, Thr6H_α_-Ile8H, Asn14H_α_-Leu16H, Leu16H_α_-Lys18H, and Asp17H_α_-Tyr19H. Medium distance (H_α_–HN *i*, *i* + 4) correlation were also observed between Ile1H_α_-Leu5H, Val4H_α_-Ile8H, Thr6H_α_-Thr10H, Val9H_α_-Lys13H, Pro11H_α_-Gln15H, Lys13H_α_-Asp17H, Asn14H_α_-Lys18H, Gln15H_α_-Tyr19H, and Leu16H_α_-Gln20H.

For AMPCry10Aa_5, sequential NOE connectivities (H_α_–HN, HN–HN, and Hb–HN) were observed for almost the entire peptide sequence, except for H_α_–HN *i*, *i* + 1 for residues Asn3, Thr6, and 13Lys, sequential HN-HN NOEs for residues Ile1, Asn3, 12Ile, 13Lys, and 14Asn, and sequential NOE connectivity (H_β_–HN *i*, *i* + 1) for residues Ile1, Asn3, Ile11, Lys13, Asn14, and Lys18. Short distance (HN–HN *i*, *i* + 2; H_α_–HN *i*, *i* + 3; and H_α_–H_β_*i, i* + 3) and medium distance (H_α_–HN *i, i* + 4) NOEs characteristic of α-helix were observed for AMPCry10Aa_5, as shown in Figure S6b. Short distance NOEs (HN–HN *i*, *i* + 2) were observed between residues Lys5H-Ser7H, IleH11-LysH13, Leu16H-Ile18H, and 17AspH-Ile19H. NOE connections (H_α_–HN *i, i* + 3) were observed between residues Val4H_α_-Ser7H, Leu8H_α_-Ile11H, Thr10H_α_-Lys13H, Ile12H_α_-Ala15H, Lys13H_α_-Leu16H, and Asp17H_α_-Gln20H. Similarly, (H_α_–H_β_*i, i* + 3) connections were observed between residues Val4H_α_-Ser7H_β_, Ile11H_α_-Asn14H_β_, and Ile12H_α_-Ala15H_β_. Additionally, NOE connections H_α_–HN *i, i* + 2 were observed between residues Asn3H_α_-Lys5H, Val4H_α_-Thr6H, Asn14H_α_-Leu16H, and Leu16H_α_-Lys18H. Finally, a medium distance NOE (H_α_–HN *i, i* + 4) was observed only in residue Ile12H_α_-Leu16H.

The Secondary Chemical Shift (SCS) analysis of the α and β carbons in AMPCry10Aa revealed positive and negative values, respectively, except for the α-carbon of the Ile1 residue, which displayed a negative value, and β-carbons for Ile1, Ser7, Ile8, Ile12, Asp17, Tyr19, and Gln20, which showed positive values. The α-hydrogens exhibited negative SCS values, except for residues Ile2 and Ile8. For AMPCry10Aa_5, the SCS analysis of the α and β carbons revealed positive and negative values, respectively, except for α-carbons of residues Ile1 and Ile2, which displayed a negative value. The α-hydrogens exhibited negative SCS values. These SCS values indicate the deviation of the chemical shift of ^13^Cα, ^13^Cβ, and ^1^Hα resonances from random coil values. The positive SCS values for the α-carbons and negative values for the β-carbons and α-hydrogens are indicative of an α-helical structure. Structure prediction suggests the presence of an α-helix for AMPCry10Aa and AMPCry10Aa_5 from Ile2 to Gln20 and Val4 to Ile19, respectively, as can be observed in the secondary structure chart (Figure S6a,b).

The dihedral angles ϕ and ψ, generated by the Dangle in the CcpNmr analysis program, were assessed as good and consistent. Each residue’s dihedral angle values were found within a single island of the Ramachandran plot, except for residue Asn3 in AMPCry10Aa_5, which appeared in two islands. All of the values were located in the allowed regions, indicating excellent stereochemistry.

**Figure 7:**
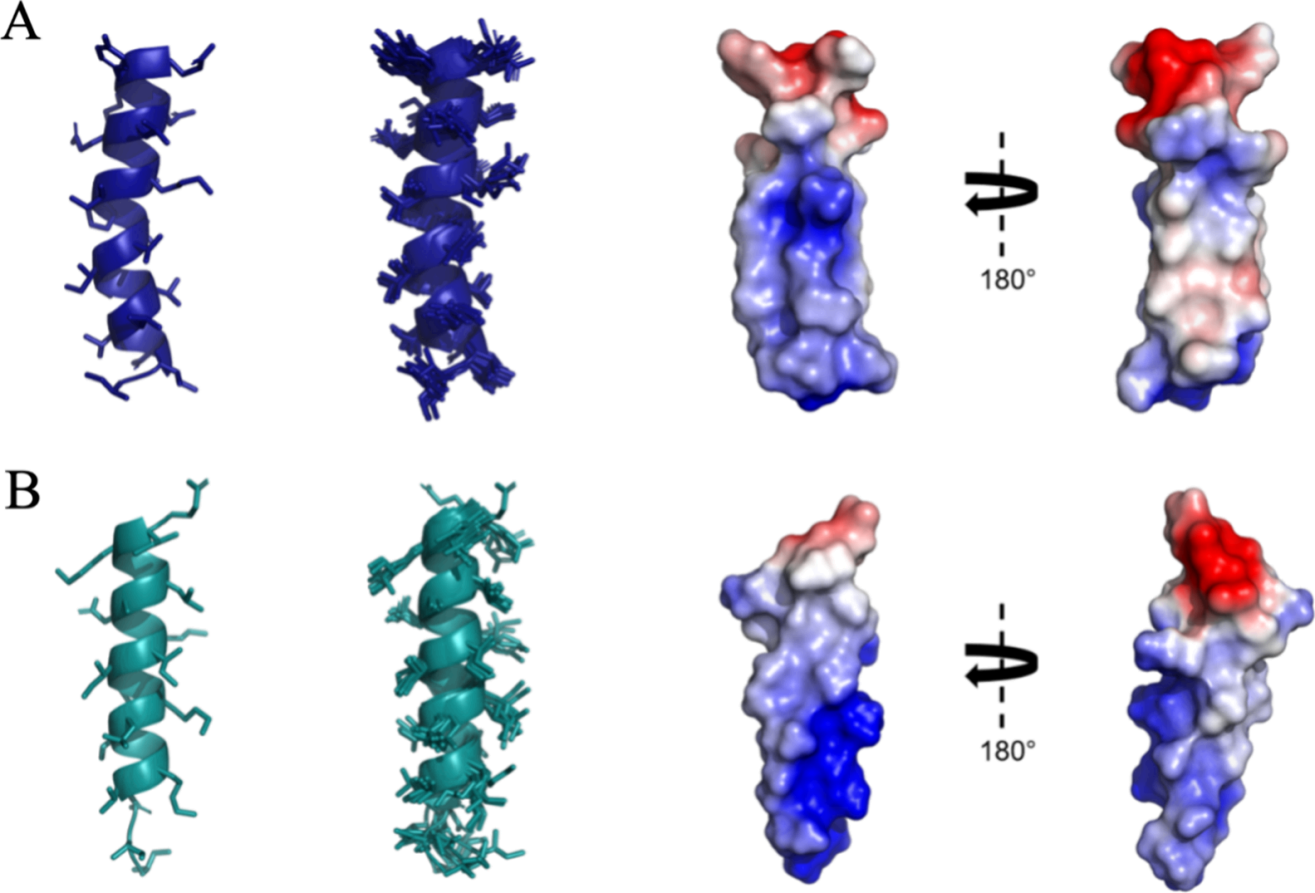
NMR structures of AMPCry10Aa and AMPCry10Aa_5. The sequence of figures, from left to right, includes: (i) the lowest energy structure represented by ribbons and (ii) the superposition of the 10 lowest energy structures of AMPCry10Aa (PDB: 8T3H) shown in blue (A) and AMPCry10Aa_5 (PDB: 8T3N) shown in green (B) in the presence of 75 mM SDS-*d*_25_ micelles. (iii) The lowest free energy model in the adaptive Poisson–Boltzmann solver (APBS) displays the electrostatic potential of the peptides, ranging from −2.5 to +2.5 *kT*/*e*, with 0° and 180° rotation (anionic regions are shown in red, cationic regions in blue, and neutral regions in white).

We observed that both peptides, AMPCry10Aa and AMPCry10Aa_5, adopt α-helix structures in an anionic environment, specifically in SDS micelles. As shown in Figure 7, the structural calculations demonstrate that the peptides assume an α-helical conformation between Ile2-Gln20 (AMPCry10Aa) and Val4-Ile19 (AMPCry10Aa_5) for the lowest energy structure in 75 mm of SDS-*d*_25_ micelles. The overlap of the ten lowest energy structures for AMPCry10Aa (PDB: 8T3H) can be seen in Figure 7A(ii), while Figure 7B(ii) illustrates the overlap for AMPCry10Aa_5 (PDB: 8T3N). For AMPCry10Aa, the α-helix structure presents a curvature in the central region, which is not observed for AMPCry10Aa_5. This effect may be a result of the presence of a proline residue (Pro11).^67^ Prolines confer a conformational restriction due to the cyclization of their side chain, with a rigidly restricted φ angle.^68^

Regarding structure convergence and RMSD values, for the AMPCry10Aa peptide, the main chain appeared to present lower rigidity, suggesting that the curvature of the helical segment can be significant in its mechanism of action. On the other hand, the side chains of AMPCry10Aa residues seemed to be significantly more rigid than those of the AMPCry10Aa_5 peptide, whose main chain gives the idea to be more rigid compared to the parent peptide, AMPCry10Aa.

The ten lowest-energy structures calculated for both peptides showed a high level of convergence, as depicted in Figure 7A, B(ii). This is evident from the low RMSD values. When aligning only the structured region of AMPCry10Aa (Ile2-Gln20), the peptide exhibits an RMSD of 0.4641 ± 0.1467 and 0.8822 ± 0.1767 Å for backbone and heavy atoms, respectively. When aligning the entire structure (Ile1-Gln20), the peptide showed RMSD of 0.4647 ± 0.1468 and 0.8804 ± 0.1763 Å for backbone and heavy atoms, respectively, demonstrating a good convergence of the structure’s ensemble. As for AMPCry10Aa_5, when aligning only the structured region (Val4-Ile19), the peptide had RMSD values of 0.1393 ± 0.0467 and 0.6122 ± 0.1329 Å for backbone and heavy atoms, respectively. When aligning the entire structure (Ile1-Gln20), the peptide had RMSD of 1.1642 ± 0.5978 and 1.5300 ± 0.5580 Å for backbone and heavy atoms, respectively,

Furthermore, as observed in Figure 7A, B(ii), the N-terminal region of AMPCry10Aa_5 exhibited high flexibility, leading to the highest RMSD value (1.5300 ± 0.5580) when aligning all heavy atoms and residues. In contrast, the N-terminal portion of AMPCry10Aa appeared more conserved, while the C-terminal regions of both peptides were more conserved.

The Ramachandran plot of the ten lowest-energy structures for AMPCry10Aa (Figure S6a) showed that 97.8% of the angles φ and ψ are located in the most favored regions, with 2.2% in allowed regions. For AMPCry10Aa_5 (Figure S6b), 94.4% of the angles are in the most favored regions, with 5.6% in the allowed regions, as indicated in Table 2. These results showed the good quality of the stereochemical of the polypeptide chain of these peptides.

The electrostatic surface of the lowest energy structures is depicted in Figure 7iii, where the anionic regions are shown in red, the cationic regions in blue, and the neutral regions in white. Although AMPCry10Aa has a net charge of +1 and AMPCry10Aa_5 has a net charge of +3, the electrostatic potential on the peptide surfaces results in a solvation potential energy of 1.83 × 10^4^ kJ/mol for AMPCry10Aa and 1.38 × 10^4^ kJ/mol for AMPCry10Aa_5. This indicates that both peptides exhibit a highly cationic nature, suggesting that electrostatic attraction, in combination with the hydrophobic interactions defined by the hydrophobic face of the peptides (Figure S7a,b), may play a crucial role in the process of membrane binding and the mechanism of action,^69^ respectively, of these peptides. As is well-known for the AMP mechanism of action, this interaction likely leads to the disordering of the phospholipid bilayer, ultimately contributing to the satisfactory antimicrobial activity exhibited by both peptides. Detailed structural statistics can be found in Table 2. Further validations, such as clash score, Ramachandran outliers, and side-chain outliers, are available in the Protein Data Bank (PDB) under PDB IDs.

### Outermost Bacterial Membrane Peptide Interaction Analysis

Several studies on mechanisms of action of synthetic antimicrobial peptides against bacteria and fungi have associated their activities with increased reactive oxygen species (ROS) production and loss of membrane integrity.^70−72^ Aiming to obtain additional information about the mechanisms of antibacterial peptides studied here, EPR spectroscopy was used to examine the peptide interactions with the bacterial peripheral membranes immediately after treatment and 24 h after peptide treatment.

**Figure 8:**
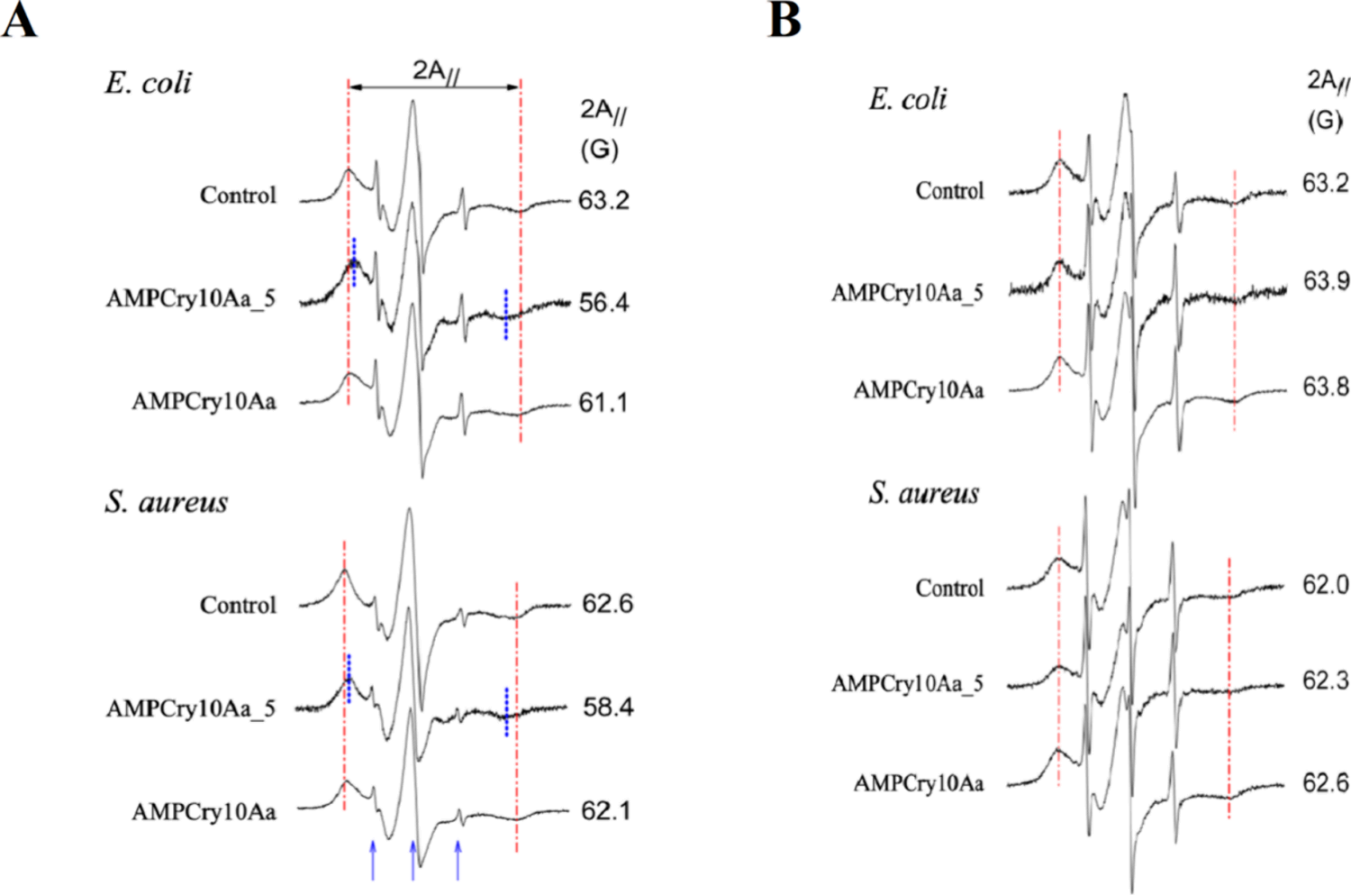
Electron paramagnetic resonance (EPR) spectra of spin label 5-DSA inserted into the outermost membrane of *E. coli* and *S. aureus* for untreated samples (controls) and treated with AMPCry10Aa and AMPCry10Aa_5. The treatment was carried out with incubation for 20 min at a rate of 1 μg of peptide for 1 × 10^7^ cells. The values of the EPR parameter 2*A*_∥_ (outer hyperfine splitting), which is given by the magnetic field separation between the first peak and the last inverted peak, are indicated for each EPR spectrum. The estimated experimental error of 2*A*_∥_ is 0.5 G. The intensity of the spectra is in arbitrary units (*Y* axis), and the total scan range of the magnetic field in each EPR spectrum was 100 G (*X*-axis). The three blue arrows indicate the positions of three resonance lines coming from spin labels that are outside the membrane, tumbling free in the aqueous solution. These narrow lines from a small fraction of free probes are present in almost all spectra and are not considered in the analyses (A). Representative EPR spectra of 5-DSA in the membrane of *E. coli* and *S. aureus* after a 24 h assay with untreated cells (controls) and cells treated with AMPCry10Aa and AMPCry10Aa_5 at a concentration of 1× MIC (4 μg·mL^–1^) and cell concentration of 1 × 10^7^ CFU·mL^–1^. Two *A*_∥_ for *S. aureus* was significantly lower than for *E. coli*, but for each bacteria there were no significant differences between the means for treated and untreated samples (*P* < 0.05) (B).

The EPR spectra (Figure 8A) indicate that the spin probe was structured in very rigid membranes, presenting 2*A*_∥_ values of ∼62 to ∼63 G, much higher than those found for the *Leishmania* parasite (∼54 G).^73−76^ Thus, it is very likely that the spin probe did not access the bacteria’s plasma membrane but was incorporated into the outermost membranes, such as the cell wall. AMPCry10Aa_5 peptide caused a large increase in fluidity in the membranes of both bacteria, having a much greater effect on *E. coli* membrane, while AMPCry10Aa parental caused a small increase in fluidity in *E. coli* membrane, with no change in the *S. aureus* membranes spin label dynamics. It is worth mentioning that AMPCry10Aa did not dissolve in the aqueous treatment solution, where it formed a suspension of aggregates, which might be the reason for an insufficient quantity in the membranes accessed by the spin-label. Pronounced increases in lipid fluidity generally create disorder in the lipid chains packaging, especially at lipid–protein interfaces, where they increase the likelihood of pore formation that could lead to electrolyte leakage and permeation of small molecules.^77^

For *S. aureus* 118, 117, and 111 samples, it was not possible to obtain the EPR spectra with the usual spin labeling method, due to the rapid reduction of the nitroxide radical present in the spin label, eliminating the EPR signal. On the bacteria periphery, there must be some powerful nitroxide-reducing agent. Nitroxide can be quickly reduced by Fe^2+^ and it is known that *S. aureus* have siderophores in their periphery, which are small molecules that are secreted by bacteria and have an exceptionally high affinity for iron.^78^

The representative EPR spectra obtained after 24 h treatment of bacteria in culture medium (Figure 8B) analysis indicated that AMPCry10Aa and AMPCry10Aa_5 peptides did not alter *E. coli* and *S. aureus* membrane fluidity when treated at concentrations corresponding to 1× MIC. Membrane rigidity probably occurs at the bacteria plasma membrane when an increased ROS formation is promoted. However, here the spin label was retained in the outermost membranes, which already have very high rigidity and must be formed by molecules that are less vulnerable to internal oxidative stress.

## Conclusions and Prospects

AMPs have a broad spectrum of activity, with the potential to avoid (less likely) antibiotic resistance mechanisms. Our study revealed that Cry10A can be used as a parental sequence to produce AMPs. Six variants were developed, analyzed, and had their antimicrobial activities tested in vitro against Gram-negative and Gram-positive bacteria. With the results obtained, we can report that AMPCry10Aa_5 was the most promising against the bacteria tested and therefore can be considered an important molecule in the future development of antibiotics. Furthermore, four of the six variants generated showed potential for at least one tested strain. In other words, when Joker combined the dermaseptin B pattern with the Cry10Aa sequence, it was successful. Therefore, both the choice of the template sequence and the method used to develop the variants were successful, obtaining possible AMPs for use against bacterial infections.

## Experimental Section

### Peptide Redesign

Initially, the sequence of the Cry10Aa protein responsible for the portion of the α-helix 3 region (IINVLTSIVTPIKNQLDKYQEFFDKWEPA) was used.^42^ Heliquest software (https://heliquest.ipmc.cnrs.fr/)^79^ was used to cut the sequence to a size of 20 amino acids and choose the best window, aiming at hydrophobicity and charge with higher values. By using the Joker algorithm,^27^ variants were generated from the sequence of 20 amino acids (IINVLTSIVTPIKNQLDKYQ) by using the pattern obtained from dermaseptin sequences (K-[ADEGNQST]-x-[AGL]-K-x-[AILV]-x(3)-A-x(3)-[AGILV]), where K and A (outside the bracket) represent identity components, the amino acids inside the bracket represent ambiguous components, the ‘X’ represents the wildcard element and the ‘3’ in parentheses represents the number of elements that are repeated. The variants generated by Joker, as well as the parental sequence, were analyzed in Heliquest software, which determines the helix’s physicochemical properties based on amino acid sequence (hydrophobic moment, charge, and average hydrophobicity).^79^ The helical-wheel projection was also generated for the variants and parental sequence, and the analysis was performed using a 20-residue window. For hydrophobicity calculation, the Eisenberg, Weiss, and Terwilliger Scale^60^ was used.

### Peptide Synthesis

The parental sequence and the six generated variants were synthesized using *N*-9-fluorenylmethyloxycarbonyl (FMOC) technology, with 95% purity, with Peptide 2.0 (USA). Matrix Assisted Laser Desorption Ionization Time of Flight (MALDI-ToF) was used to validate the molecular mass of the peptides on a mass spectrometer UltraflexMALDI-TOF III (Bruker Daltonics) (Table S4).

### Determination of Minimum Inhibitory Concentration (MIC) and Minimum Bactericidal Concentration (MBC)

The antimicrobial activity of the peptides was assessed by estimating the minimum inhibitory concentration (MIC), which was obtained using the broth microdilution technique, according to the NCSLA guidelines, as reported by Wiegand et al.^80^*Staphylococcus aureus* 111, *Staphylococcus aureus* 117, *Staphylococcus aureus* 118, and *Acinetobacter baummanii* 003324845 were obtained from the Culture Collection of Universidade Católica de Brasília and *Staphylococcus aureus* 25923, *Escherichia coli* 8739, *Enterobacter cloacae* 49141, *Enterococcus facallis* 29212, *Klebsiella pneumonia* 13883, and *Pseudomonas aeruginosa* 27853 were obtained from the American Type Culture Collection (ATCC). Bioassays were performed in microdilution plates. The strains were cultured overnight at 37 °C in a Mueller–Hinton broth. MIC measurements were performed using 1 × 10^6^ CFU·mL^–1^ and serial dilution of the peptide variants at an initial concentration of 256 μg·mL^–1^. As a negative control, bacterial culture at a concentration of 1 × 10^6^ was used as well as bacteria with antibiotics (chloramphenicol) as positive control. The variants were tested against *Staphylococcus aureus* and *Escherichia coli* in biological triplicate. The plate was incubated at 37 °C for 24h and the reading was performed after 24 h in the Biotek spectrophotometer (PowerWaveTM HT Microplate Reader) at a wavelength of 595 nm. After MIC determination, aliquots of 10 μL from all the wells which showed no visible bacterial growth were removed and plated in Mueller-Hinton agar and incubated for 24 h at 37 °C. After incubation, the colonies were counted to obtain the minimal bactericidal concentration end point. The MBC is the concentration at which no colonies are observed.^81^

### Time-Kill Kinetics Assays

Time-kill kinetics were performed using the parental peptide AMPCry10Aa, as well as AMPCry10Aa_1 and AMPCry10Aa_5, against *E. coli* ATCC, *S. aureus* 111 and *S. aureus* ATCC strains, as previously described.^82,83^ Mid-logarithmic growing bacteria was diluted to 5 × 10^6^ CFU·mL^–1^ in PBS (10 mM potassium phosphate, 100 mM NaCl, pH 7.3). Afterward, bacterial cultures were exposed to MBC determined for the peptides. 100 μL aliquots were removed every 10 min for 120 min, then diluted (1:10, three subsequent dilutions) in saline (0.9%), and seeded (50 μL) on Mueller Hinton agar plates. Colony counting was performed manually after 18 h of incubation at 37 °C. Bacterial growth under the same conditions without the presence of the peptide was evaluated to be used as a control of cell viability.

### Cytotoxicity Assay

To evaluate the cytotoxicity of the peptides, a 3-(4,5-dimethylthiazolyl-2)-2,5-diphenyltetrazolium bromide (MTT) assay was conducted.^84,85^ Raw 264.7 macrophage cell line was maintained under sterile conditions. Cultured in DMEM (SIGMA) with 10% fetal bovine serum (SIGMA), the cells were incubated at 37 °C in an environment of over 95% humidity and 5% CO_2_ within a 75 cm^2^ (KASVI) culture flask. Upon reaching 90% confluence, cells were detached, centrifuged at 970*g* for 5 min at room temperature, and then seeded at 2 × 10^5^ cells per well in a 96-well plate. Peptides, ranging from concentrations of 2–128 μg·mL^–1^, were added and incubated at 37 °C for 24 h. After the supernatant was discarded, a 5 mg·mL^–1^ MTT solution (SIGMA) diluted in PBS was applied to each well. The covered plate was incubated for 4 h at 37 °C in darkness. Formazan crystals were solubilized using a hydrochloric acid and isopropyl alcohol solution, and cell viability percentages were determined at 540 nm using a microplate reader (Thermo Scientific Multiskan Britain). The results were calculated in relation to the untreated cell control.

### Peptide Stability Assay

The stability of peptides AMPCry10Aa, AMPCry10Aa_1, and AMPCry10Aa_5, against serum proteases was evaluated as previously described.^86,87^ Aliquots of filtered human serum from male AB plasma from Sigma-Aldrich were mixed with each peptide to a final concentration of 256 μM in a 5:1 v/v ratio. Each aliquot was then incubated at 37 °C, and 200 μL was collected for analysis of peptide degradation at 0, 2, 4, 6, 12, and 24 h. The samples were analyzed by reversed-phase high-performance liquid chromatography (RP-HPLC) with a Shimadzu LC system (Kyoto, Japan) using LC Solution software. A gradient method and a Venusil ASB C18 column (250 mm × 4.6 mm, 5 μm, Bonna-Agela Technologies) with an injection volume of 15 μL were used. A segmented gradient was applied with a constant flow rate of 1.0 mL min^–1^ and PDA detection at 216 nm using water and acetonitrile eluents as the mobile phase. The peptide amount was quantified by subtracting the relative area of each analyzed time peak from the initial control peak area. All assays were performed in triplicate.

### Temperature Stability

To analyze the thermal stability of peptides, AMPCry10Aa, AMPCry10Aa_1, and AMPCry10Aa_5 peptides were incubated at different temperatures (37, 50, and 100 °C) for 30 min. The untreated peptides were used as a control. The antibacterial activity of treated peptides against *S. aureus* 111, *S. aureus* ATCC 25923, and *E. coli* ATCC 8739 was determined by MIC assay as described above. All assays were performed in triplicate.

### Molecular Modeling

Molecular modeling of the Cry10Aa protein, the parental peptide, and its variants was performed using the Alphafold2 tool.^88,89^ For smaller proteins, AlphaFold 2 has generally shown high accuracy, but considering the specific characteristics of the studied peptides, validation of the predictions with experimental methods for structure confirmation. Therefore, after performing all of the modeling, the models were then validated using software that estimates the quality of the generated model. The types of software used were: QMEAN, which evaluates the quality of the model based on comparisons with similar structures already elucidated;^90^ ProSA (https://prosa.services.came.sbg.ac.at/prosa.php), which computes an overall quality score for a certain input structure and if this score falls outside a characteristic range for native proteins, the structure is likely to contain errors^91,92^; and PROCHECK (https://servicesn.mbi.ucla.edu/PROCHECK/), which compares stereochemical properties acquired from well-refined high-resolution structures to the geometry of residues in a specific protein structure using the Ramachandran plot.^93^

### Nuclear Magnetic Resonance (NMR) Spectroscopy

Solution NMR spectroscopy for the peptides AMPCry10Aa (IINVLTSIVTPIKNQLDKYQ-NH2) and AMPCry10Aa_5 (IINVKTSLKTIIKNALDKIQ-NH2) was performed using 75 mM deuterated SDS (SDS-*d*_25_, Cambridge Isotope Laboratories, USA) at pH 4.6. The peptide solutions were prepared at 1.5 mM in 250 μL of H_2_O/D_2_O (90/10, v/v, Cambridge Isotope Laboratories, USA). 3-(Trimethylsilyl)-1-propanesulfonic acid-d_6_ sodium salt (DSS-*d*_6_, Sigma-Aldrich, USA) at 0.05% was used as an internal chemical shift reference. All spectra were acquired at 298 K on a Bruker Avance III 500 spectrometer equipped with a 5 mm broad-band inverse (BBI) probe. ^1^H–^1^H TOCSY experiments were run using the dipsi2gpph19^94^ pulse sequence with 96 transients of 4096 × 512 points (F2, F1) and spinlock mixing time of 70 ms. The ^1^H–^1^H NOESY spectra were acquired using the noesygpph19^94^ pulse sequence with 120 transients of 4096 × 512 points and a mixing time of 150 ms. ^1^H–^13^C HSQC experiments were acquired using the hsqcetgp and hsqcedetgp^95^ pulse sequence with 138 and 69 transients of 4096 × 512 points, respectively. All 2D NMR data were processed by using Bruker TopSpin 3.6.3 and analyzed by using CcpNMR Analysis 2.5.2 software. The Wuthrich method^96^ was employed to assign spin systems based on the observed ^1^H resonances in TOCSY and NOESY spectra. ^1^H–^13^C HSQC heteronuclear spectra were used to assist in the assignment of spin systems and confirm the assignment of chemical shifts.

### Structure Determination

The calculation and refinement of the three-dimensional structures were performed using ARIA^97−99^ software version 2.3 with the compilation of the CNS program^100^ and by molecular dynamics simulated annealing protocol (MDSA).^101^ The volumes, classified as strong, medium, and weak and obtained by NOE peak correlations from the NOESY spectra, were semiquantitatively converted into distance restraints using 1.72, 3.2, and 8.0 Å as a lower limit, reference distance, and upper distance limit, respectively.

The dihedral angle restraints were determined from the chemical shifts of ^1^HN, ^1^H_α_, ^13^C_α_, and ^13^C_β_ by using the DANGLE algorithm^102^ from CcpNMR Analysis software.^103^ Both distance and dihedral angle restraints were employed as data input from the CCPN^104^ in the calculation of ARIA 2.3 software. Two hundred structures were generated for each iteration in a 9-iteration protocol (it0 until it8), followed by 20,000 steps of simulated annealing at 1000 K and a subsequent decrease in temperature in 15,000 steps in the first slow-cool annealing stage. For the last iteration, the ten lowest energy structures were selected and subjected to refinement using the water refinement protocol.^105^ Structures were visualized using the PyMOL^106^ and UCSF Chimera software.^107^ The ten lowest energy conformations represented the ensemble of structures. The validation and structural analysis of the calculated structures were performed by using various quality parameters. The convergence of the structures was assessed by using root-mean-square deviation (RMSD) values. The stereochemical quality was evaluated using PROCHECK^108^ through the Ramachandran diagram. The fold quality was determined using *Z*-scores obtained from ProSA (Protein Structure Analysis) server.^92,109^ The dihedral angles and covalent forces were evaluated using the *G*-factor in the PDBsum server.^110^ Additionally, the model was evaluated using QMean in the SWISS-MODEL server,^111^ which compared it with previously elucidated similar structures. Furthermore, the structures were required to have a minimum total energy during the calculation.

### Solvation Potential Energy Calculation

The solvation potential energy calculations were performed for the lowest energy tridimensional theoretical structures developed by molecular modeling and the ones elucidated by solution NMR spectroscopy (AMPCry10Aa and AMPCry10Aa_5). The PDB 2PQR service was used to convert.pdb files to.pqr files using the AMBER force field.^112^ PDB 2PQR also defined the grid dimensions for the APBS computation. On APBS, the solvation potential energies were estimated.^113,114^ The surface was visualized using PyMOL’s APBS plugin.

### Electron Paramagnetic Resonance (EPR) Spectroscopy

Peptides' immediate effects on bacteria’s outermost membrane fluidity were analyzed by EPR spectroscopy. The bacteria in culture (1 × 10^9^ CFU) were centrifuged and resuspended in 50 μL of PBS containing 100 μg of peptide. After 20 min of incubation, the sample was spin-labeled for the EPR experiment. To incorporate the spin label into the bacterial membranes, a 5 mg·mL^–1^ ethanolic solution containing the spin label 5-doxyl stearic acid (5-DSA, Sigma-Aldrich, St. Louis, MO, USA) was added to each sample. Immediately after spin labeling, the sample was transferred to a 1 mm i.d. capillary tube, which was flame-sealed on one side. The capillary was centrifuged at 25,000 × *g* for 5 min, and the cell pellet of approximately 2 mm was placed in the center of the resonance cavity. Moreover, peptide effects after 24 h treatment, to measure antimicrobial activity, was carried out in a 24-well plate, using 1 × 10^7^ CFU·mL^–1^ and peptide concentration of 1× MIC. After 24 h of incubation, the treated and untreated samples were centrifuged at 25,000 × *g* and the supernatant was completely removed. Cells were resuspended in 50 μL of PBS and spin-labeled as described above for EPR measurements.

The EPR spectra were recorded by using a Bruker EPR EMXplus spectrometer (Bruker BioSpin GmbH, Rheinstetten, Germany). The instrumental settings were as follows: microwave power, 2 mW; microwave frequency, 9.45 GHz; modulation frequency, 100 kHz; modulation amplitude, 1.0 G; magnetic field scan, 100 G; sweep time, 168 s; and sample temperature, 25 ± 1 °C.

### Statistical Analysis

Each experiment was conducted at least three independent times and the data are expressed as mean and standard deviation (SD). The means were compared through a one-way analysis of variance (ANOVA). Tukey’s test was used to identify significant differences (*P* < 0.05) between the means of the different treatments.

## Supporting Information Available

Additional experimental details, including tables and figures (PDF)
Figures showing antibacterial activity, structure analysis, stability analysis, cytotoxicity analysis, and mechanism of action analysis (PDF)
