# Supplementary material for: Anti-Staphy Peptides Rationally Designed from Cry10Aa Bacterial Protein
Source: ACS Omega. 2024 Jun 19;9(27):29159–74. doi: 10.1021/acsomega.3c07455 (PMC11238290; doi:10.1021/acsomega.3c07455)

Anti-Staphy peptides rationally designed from Cry10Aa bacterial protein

Thuanny Borba Rios[1,2], Mariana Rocha Maximiano[1,2], Fabiano Cavalcanti Fernandes[2], Gabriella Cavalcante Amorim[2,3], William Farias Porto[4], Danieli Fernanda Buccini[1], Valentina Nieto Marín[1], Gabriel Cidade Feitosa [2, 6] Carlos Daniel Pereira Freitas[5], Juliana Bueno Barra[5], Antonio Alonso[7], Maria Fátima Grossi de Sá[2,3], Luciano Morais Lião*[5], Octávio Luiz Franco*[1,2]

1 - S-Inova Biotech, Programa de Pós-Graduação em Biotecnologia Universidade Católica Dom Bosco, Av. Tamandaré, 6000 – Jardim Seminario, Campo Grande — MS, 79117-900, Brazil.

2 - Centro de Análises Proteômicas e Bioquímicas, Programa de Pós-Graduação em Ciências Genômicas e Biotecnologia Universidade Católica de Brasília, St. de Grandes Áreas Norte 916 — Asa Norte, Brasília — DF, 70790-160, Brazil.

3 - Embrapa Recursos Genéticos e Biotecnologia, Parque Estação Biológica, PqEB, Av. W5 Norte – Asa Norte, Brasília — DF, 70770-917, Brazil.

4 - Porto Reports, Brasília – DF, 70770-917, Brazil.

5 - Laboratório de RMN, Instituto de Química, Universidade Federal de Goiás, Goiânia — GO, 74690-900, Brazil.

6- Universidade de Brasília, Pós-Graduação em Patologia Molecular, Campus Darcy Ribeiro, Brasília/DF, Brazil, 70910-900.

7 - Instituto de Física, Universidade Federal de Goiás, Goiânia — GO, 74690-900, Brazil

Table S1. Validation of the generated models.

Variant	Ramachandran chart		QMean	ProSA Z-score
	Residues in favorable regions	Residues in permitted regions		
AMPCry10Aa	94.10%	5.90%	-0.46	0.35
AMPCry10Aa_1	94.40%	5.60%	-0.01	-0.73
AMPCry10Aa_2	100.00%	0.00%	-0.16	-0.32
AMPCry10Aa_3	100.00%	0.00%	-0.71	-1.07
AMPCry10Aa_4	70.60%	23.50%	-1.67	-0.79
AMPCry10Aa_5	88.90%	11.10%	0.56	-1.28
AMPCry10Aa_6	88.20%	5.90%	0.95	-1.27

Table S2. Chemical shift of AMPCry10Aa in 75 mM of SDS-d_{25} micelles.

Residue	H	Hα	Hβ	Hγ	Hδ	Hε	Cα	Cβ	Cγ	Cδ	Cε
**Ile1**	NF	4.07	2.00	1.01, 1.31, 1.61	NF	-	60.56	39.18	16.74, 27.41	NF	-
**Ile2**	8.05	4.21	1.98	1.01, 1.28, 1.59	NF	-	61.79	38.60	17.83, 27.30	NF	-
**Asn3**	8.49	4.63	2.83, 2.96	-	6.87, 7.62	-	54.91	38.81	NF	-	-
**Val4**	8.13	3.96	2.23	1.00, 1.05	-	-	63.51	31.96	21.25, 21.63	-	-
**Leu5**	7.83	4.18	1.70, 1.87	1.57	0.94, 0.94	-	56.34	41.32	27.08	25.11	-
**Thr6**	7.98	4.05	4.26	1.28	-	-	65.56	68.79	22.02	-	-
**Ser7**	8.00	4.42	3.97, 3.97	NF	-	-	61.17	64.79	-	-	-
**Ile8**	7.53	4.26	2.05	0.94, 1.31, 1.31	NF	-	62.63	39.18	17.74, 27.22	NF	-
**Val9**	7.84	3.71	2.23	1.02, 1.10	-	-	66.28	31.83	21.83, 22.61	-	-
**Thr10**	8.06	4.45	4.09	1.25	-	-	66.03	68.91	21.80	-	-
**Pro11**	-	4.45	1.80, 2.45	2.04, 2.23	3.50, 3.75	-	66.89	31.25	28.03	49.95	-

**Ile12**	7.21	3.67	2.08	0.90, 1.30, 1.30	NF	-	65.71	39.12	17.33, 27.17	NF	-
**Lys13**	8.65	3.88	1.92, 2.00	1.44, 1.64	1.75, 1.86	2.99, 2.99	60.26	32.18	25.66	27.04	42.11
**Asn14**	8.47	4.48	2.83, 3.00	-	6.82, 7.58	-	55.76	38.21	NF	-	-
**Gln15**	7.91	4.20	2.20, 2.27	2.21, 2.72	-	NF	57.66	27.95	32.93	NF	-
**Leu16**	8.41	4.16	1.76, 1.86	NF	0.92, 0.92	-	56.29	41.55	NF	25.31, 25.31	-
**Asp17**	8.30	4.50	2.85, 2.95	-	NF	-	55.85	39.05	NF	-	-
**Lys18**	7.65	4.05	1.82, 1.82	1.25, 1.45	1.65, 1.65	2.93, 2.93	58.40	32.49	24.93	29.43	42.29
**Tyr19**	7.82	4.44	3.00, 3.22	-	7.19, 7.19	6.84, 6.84	59.49	39.09	NF	133.19	118.16
**Gln20**	8.05	4.18	2.05, 2.14	2.39, 2,50	-	6.76, 7.27	57.95	29.63	34.23	NF	-
**NH_2**	6.96, 7,02	-	-	-	-	-	-	-	-	-	-

"-" is used to indicate that the resonance is not applicable to the spin system.
"NF" represents "Not Found" and is used to indicated that the chemical shift for the resonance atom could not to be determined.

Table S3. Chemical shift of AMPCry10Aa_5 in 75 mM of SDS-d_{25} micelles.

Residue	H	Hα	Hβ	Hγ	Hδ	Hε	Cα	Cβ	Cγ	Cδ	Cε
**Ile1**	NF	4.03	1.99	1.04, 1.60, 1.60	NF	-	60.38	39.15	16.70, 26.82	NF	-
**Ile2**	8.02	4.21	1.90	0.95, 1.18, 1.18	NF	-	61.49	39.28	17.51, 27.05	NF	-
**Asn3**	8.40	4.85	2.78, 3.01	-	6.85, 7.61	-	NF	38.70	NF	-	-
**Val4**	8.40	3.74	2.21	0.95, 1.04	-	-	65.58	31.95	21.57, 22.18	-	-
**Lys5**	8.26	3.93	1.95, 1.95	1.38, 1.38	1.80, 1.80	3.03, 3.03	60.59	32.13	25.86	29.37	42.16
**Thr6**	7.99	4.04	4.17	1.25	-	-	65.30	68.69	21.80	-	-
**Ser7**	8.13	4.39	3.91, 4.05	NF	-	-	61.77	63.05	-	-	-
**Leu8**	8.44	4.08	1.57, 1.57	1.86	0.90	-	58.06	41.76	27.07	23.95	-

**Lys9**	8.14	3.83	1.97, 1.97	1.64, 1.64	1.78, 1.78	2.97, 2.97	60.94	32.02	25.83	29.38	42.10
**Thr10**	7.81	3.86	4.39	1.27	-	-	66.86	68.95	21.84	-	-
**Ile11**	7.93	3.84	2.00	0.97, 1.27, 1.27	0.89	-	65.32	38.49	17.49, 28.52	13.69	-
**Ile12**	8.27	3.67	1.97	0.94, 1.16, 1.16	0.83	-	65.18	37.39	17.55, 27.12	13.09	-
**Lys13**	8.32	3.93	2.00, 2.00	1.49, 1.49	1.66, 1.66	NF	59.83	32.01	25.04	28.94	NF
**Asn14**	8.34	4.48	2.83, 3.05	-	6.84, 7.61	-	55.82	37.66	-	-	-
**Ala15**	8.30	4.13	1.56	-	-	-	55.43	18.27	-	-	-
**Leu16**	8.56	4.03	1.66, 1.66	1.87	0.91	-	57.90	41.47	27.03	25.17	-
**Asp17**	8.28	4.48	2.89, 3.05	-	NF	-	55.77	38.56	NF	-	-
**Lys18**	7.80	4.14	2.04, 2.04	1.54, 1.62	1.74, 1.74	3.02, 3.02	58.52	32.57	25.08	29.21	42.17
**Ile19**	7.88	3.97	2.00	0.99 1.28, 1.28	0.90	-	63.50	39.15	17.48, 26.83	13.69	-
**Gln20**	8.11	4.21	2.11, 2.11	2.38, 2,53	-	6.74, 7.23	56.72	29.57	34.48	NF	-
**NH_2**	7.16, 7,09	-	-	-	-	-	-	-	-	-	-

"-" is used to indicate that the resonance is not applicable to the spin system.
"NF" represents "Not Found" and is used to indicated that the chemical shift for the resonance atom could not to be determined.

Table S4. Sequence of peptides and molecular mass obtained by Matrix Assisted Laser Desorption Ionization.

Peptides	Sequences	Molecular mass
**AMPCry10Aa**	IINVLTSIVTPIKNQLDKYQ	2299.74
**AMPCry10Aa_1**	KDNLKTIIVTAIKNILDKYQ	2330.79
**AMPCry10Aa_2**	IKNVLKSIVTPAKNQLDKYQ	2299.74
**AMPCry10Aa_3**	IIKDLLKIVTPIANQLIKYQ	2323.89
**AMPCry10Aa_4**	IINKDTLKVIPIKAQLDIYQ	2325.82
**AMPCry10Aa_5**	IINVKTSLKTIIKNALDKIQ	2252.77
**AMPCry10Aa_6**	IINVLKSILKPIKNQADKYI	2310.85

Figure S1. Heatmap of peptide functional analyses. Minimum inhibitory concentration (MIC) and minimum bactericidal concentration (B) analyses for parental peptide and its variants against and *Acinetobacter baummanii* 003324845, *Enterobacter cloacae* 49141 ATCC, *Enterococcus facallis* 29212 ATCC, *Klebsiella pneumonia* 13883 ATCC, and *Pseudomonas aeruginosa* 27853 ATCC. Values were expressed in µg.mL-1.

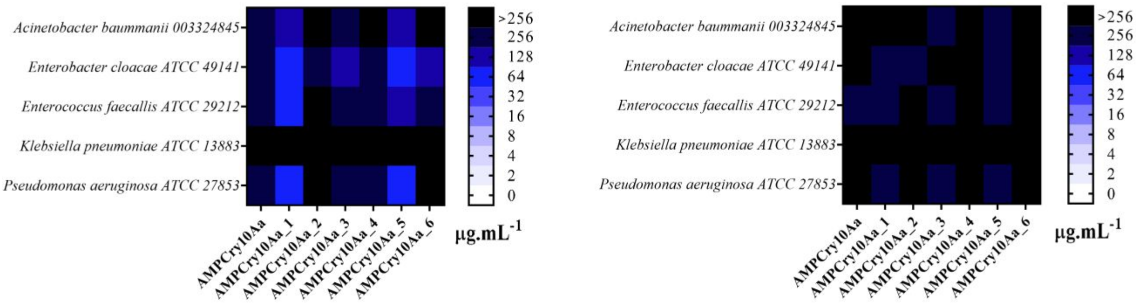

Figure S2. Stability of peptide AMPCry10Aa in serum monitored by HPLC. (A-B) Chromatograms represent the negative control of serum and peptide alone. (C-D) Chromatograms represent the mixture of serum and the peptide at 0 and 2h. The red arrow indicates the peptide0 signal detection.

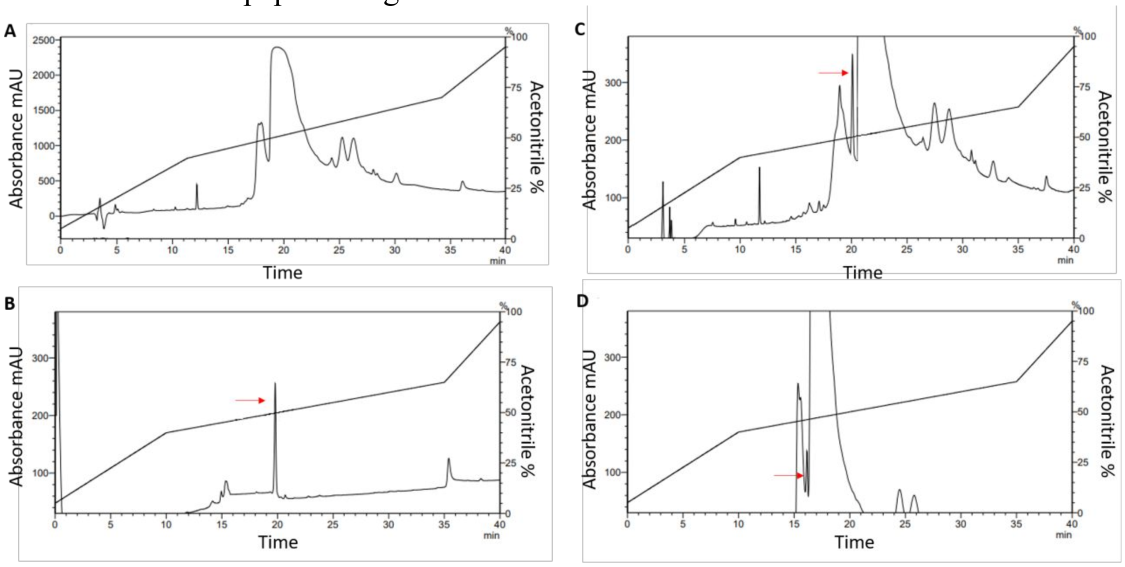

Figure S3. Stability of peptide AMPCry10Aa_1 in serum monitored by HPLC. (A-B) Chromatograms represent the negative control of human serum and peptide alone. (C-F) Chromatograms represent the mixture of human serum and the peptide at 0, 2, 4 and 6h. The red arrow indicates the peptide signal detection.

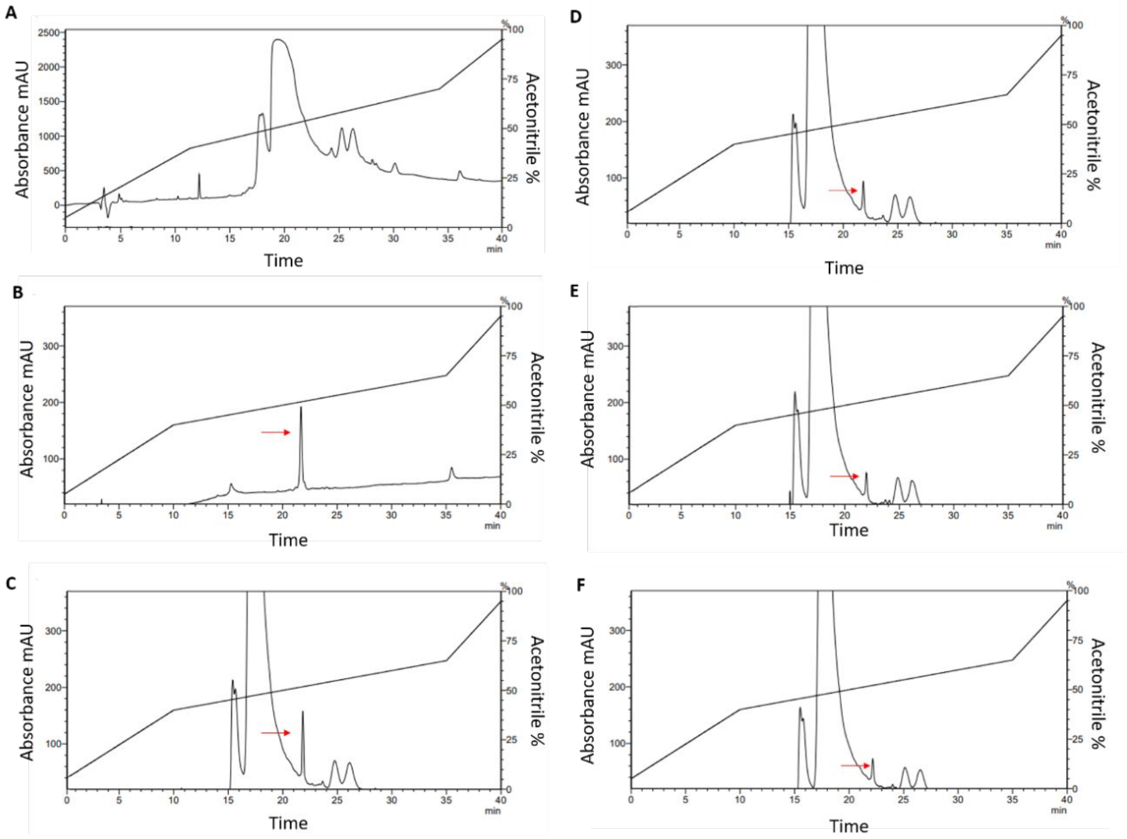

Figure S4. Stability of peptide AMPCry10Aa_5 in serum monitored by HPLC. (A-B) Chromatograms represent the negative control of 25% human serum and AMPCry10Aa_5 alone, respectively. (C-G) Chromatograms represent the mixture of 25% human serum and the peptide at 0, 2, 6, and 12h. The red arrow indicates the peptide signal detection.

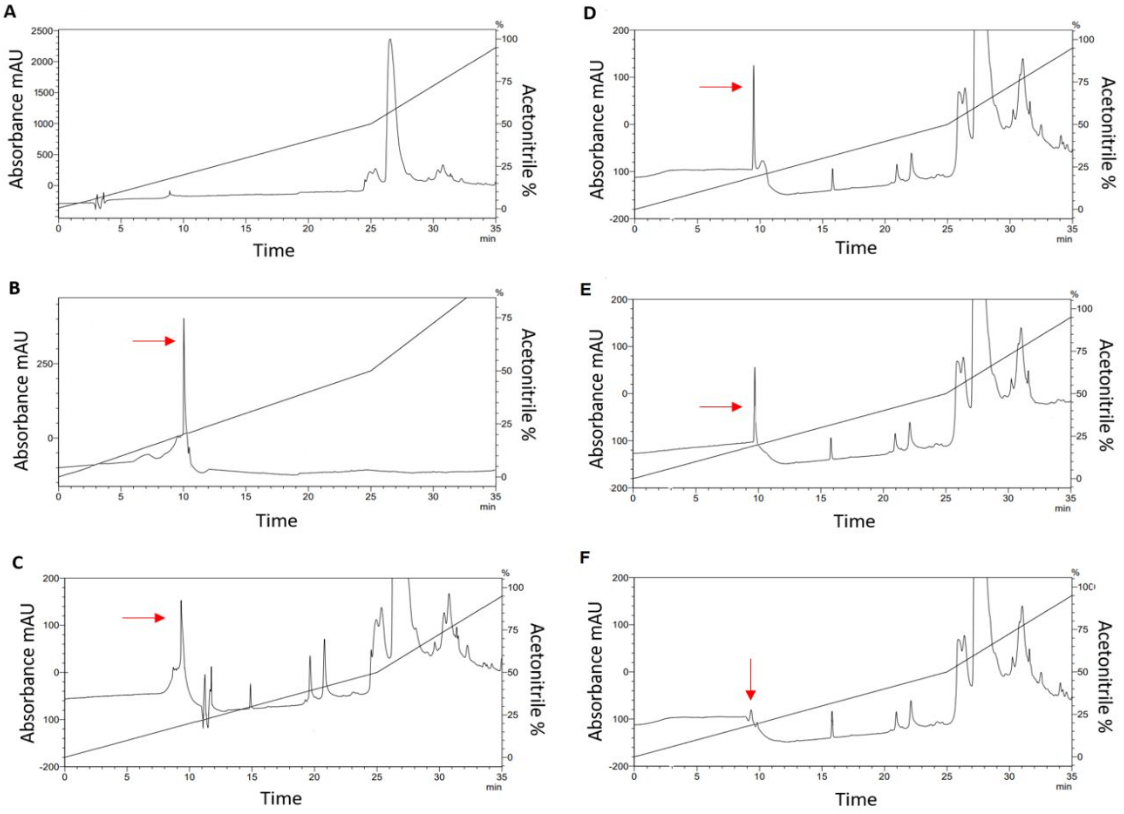

Figure S5. Peptide toxicity potential evaluated against murine macrophages (RAW 264.7 cells). Cell viability values were expressed in percentages. Bars represent the mean ± standard deviation values.

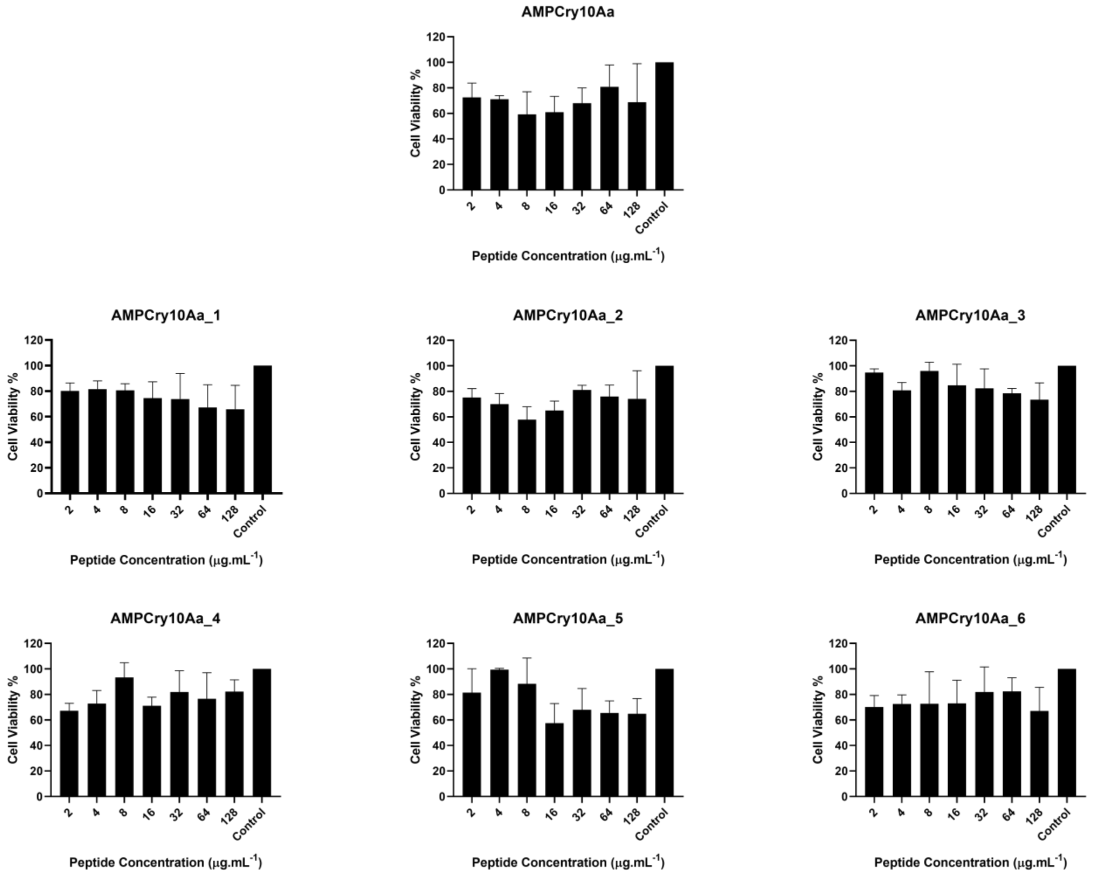

Figure S6. Sequential, short and medium-range NOE connectivity for AMPCry10Aa **(a)** and AMPCry10Aa_5 **(b)** in 75 mM of SDS-d_{25} micelles. The sequence is indicated at the top using the one-letter code, where "X" represents the C-terminal amidation. The chart illustrates the NOE connectivity, SCS values for ^{13}Cα, ^{13}Cβ and ^{1}Hα resonances, and the predicted secondary structure. The Ramachandran diagram shows the dihedral angles for the 10 lowest-energy solution NMR structures of AMPCry10Aa and AMPCry10Aa_5.

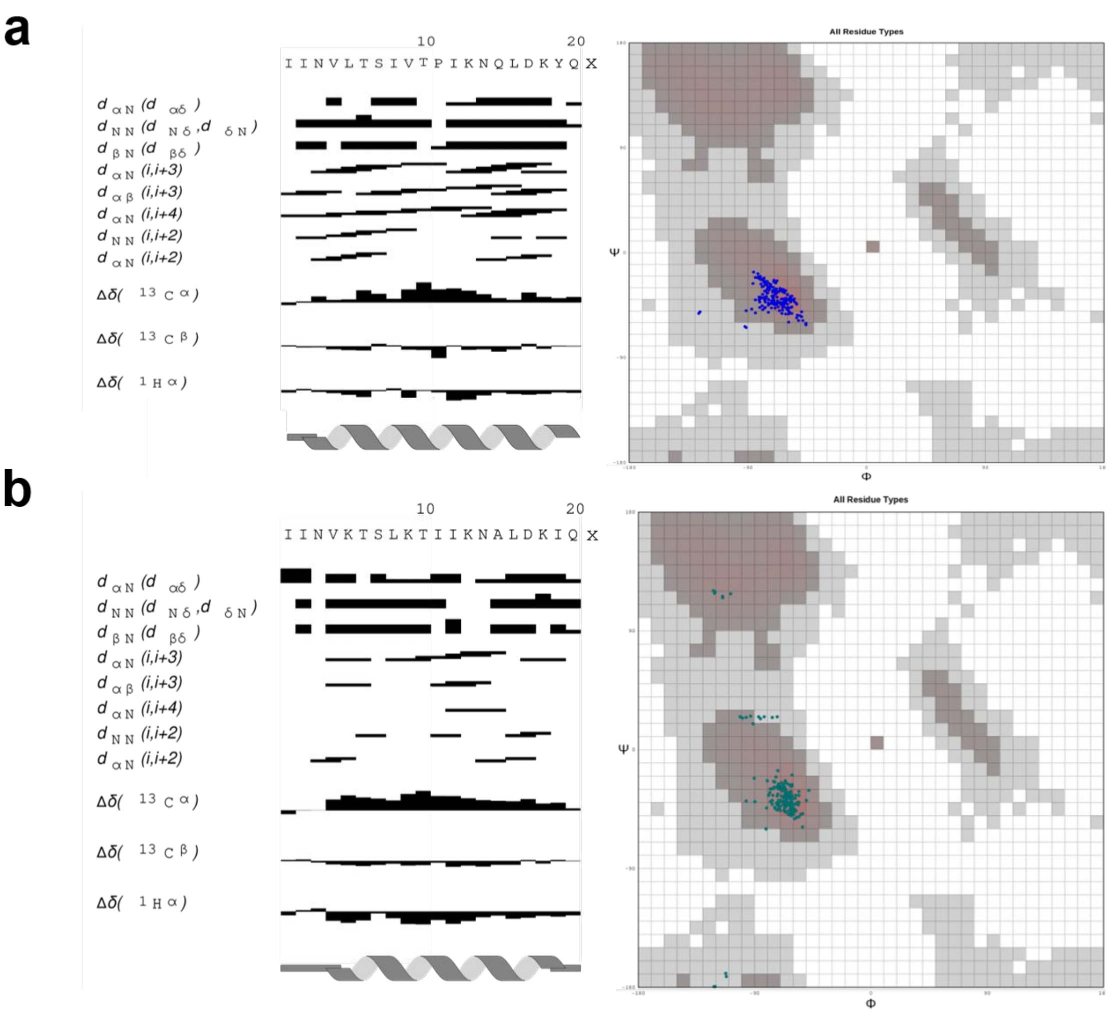
a
10
20
I I N V L T S I V T P I K N Q L D K Y Q X
All Residue Types
Ψ
Φ
b
10
20
I I N V K T S L K T I I K N A L D K I Q X
All Residue Types
Ψ
Φ

Figure S7. Distribution of prediction amino acid residues by *HeliQuest* and Front view of the inside of the α-helix, showing the hydrophobic and hydrophilic face, hydrophobic residues are in blue for the AMPCry10Aa (a) and green for AMPCry10Aa_5 (b).

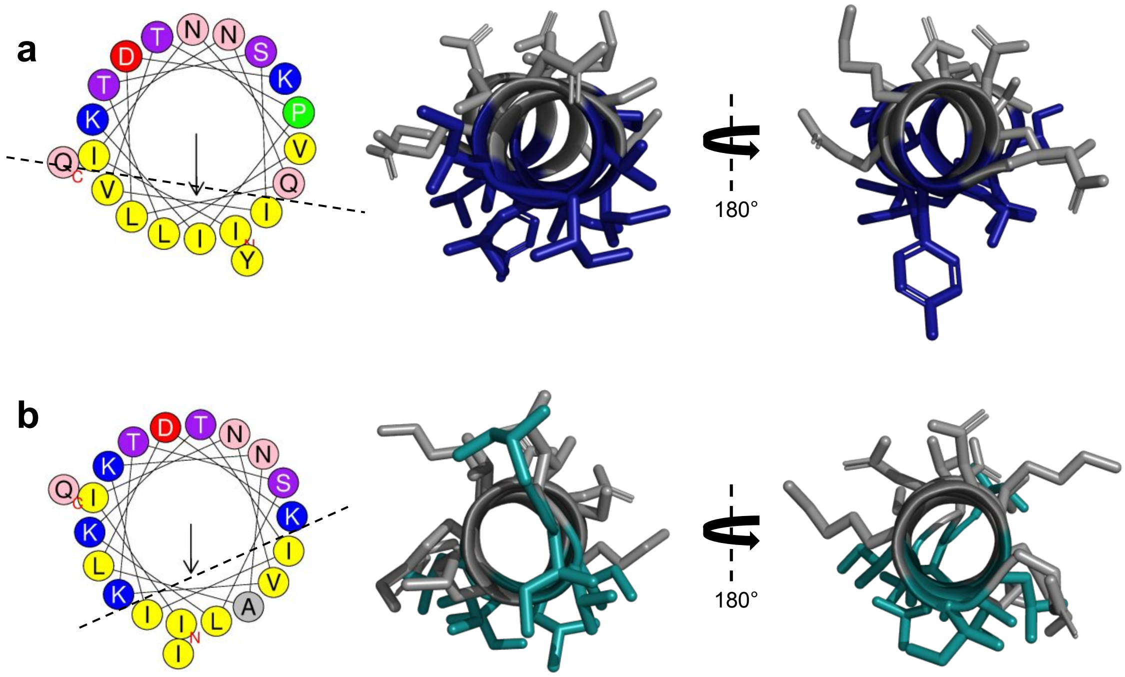

Supplement: Supplementary file 1 — ao3c07455_si_001.pdf [file ao3c07455_si_001.pdf]
