# Supplementary figures and images for: Anti-Staphy Peptides Rationally Designed from Cry10Aa Bacterial Protein
Source: ACS Omega. 2024 Jun 19;9(27):29159–74. doi: 10.1021/acsomega.3c07455 (PMC11238290; doi:10.1021/acsomega.3c07455)

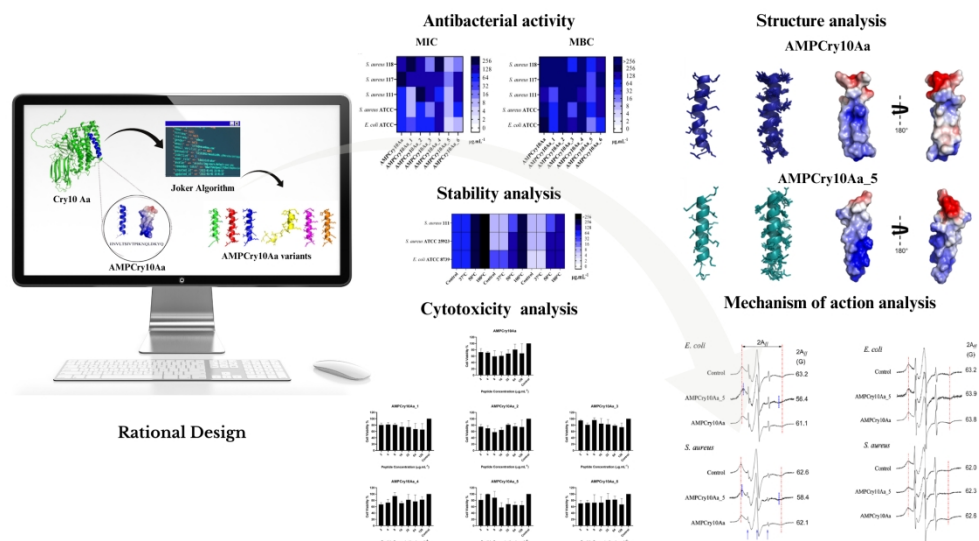

508x285mm (120 x 120 DPI)

Supplement: Supplementary file 2 — ao3c07455_si_002.pdf [file ao3c07455_si_002.pdf]
